# Essential Role of the ESX-5 Secretion System in Outer Membrane Permeability of Pathogenic Mycobacteria

**DOI:** 10.1371/journal.pgen.1005190

**Published:** 2015-05-04

**Authors:** Louis S. Ates, Roy Ummels, Susanna Commandeur, Robert van der Weerd, Marion Sparrius, Eveline Weerdenburg, Marina Alber, Rainer Kalscheuer, Sander R. Piersma, Abdallah M. Abdallah, Moataz Abd El Ghany, Alyaa M. Abdel-Haleem, Arnab Pain, Connie R. Jiménez, Wilbert Bitter, Edith N.G. Houben

**Affiliations:** 1 Department of Medical Microbiology and Infection Control, VU University Medical Center, Amsterdam, the Netherlands; 2 Institute for Medical Microbiology and Hospital Hygiene, Heinrich-Heine-University Düsseldorf, Düsseldorf, Germany; 3 Department of Medical Oncology, OncoProteomics Laboratory, VU University Medical Center, Amsterdam, the Netherlands; 4 Biological and Environmental Sciences and Engineering (BESE) division, King Abdullah University of Science and Technology (KAUST), Thuwal, Kingdom of Saudi Arabia; 5 Section Molecular Microbiology, Amsterdam Institute of Molecules, Medicine & Systems, Vrije Universiteit Amsterdam, Amsterdam, the Netherlands; University of Geneva Medical School, SWITZERLAND

## Abstract

Mycobacteria possess different type VII secretion (T7S) systems to secrete proteins across their unusual cell envelope. One of these systems, ESX-5, is only present in slow-growing mycobacteria and responsible for the secretion of multiple substrates. However, the role of ESX-5 substrates in growth and/or virulence is largely unknown. In this study, we show that *esx-5* is essential for growth of both *Mycobacterium marinum* and *Mycobacterium bovis*. Remarkably, this essentiality can be rescued by increasing the permeability of the outer membrane, either by altering its lipid composition or by the introduction of the heterologous porin MspA. Mutagenesis of the first nucleotide-binding domain of the membrane ATPase EccC_5_ prevented both ESX-5-dependent secretion and bacterial growth, but did not affect ESX-5 complex assembly. This suggests that the rescuing effect is not due to pores formed by the ESX-5 membrane complex, but caused by ESX-5 activity. Subsequent proteomic analysis to identify crucial ESX-5 substrates confirmed that all detectable PE and PPE proteins in the cell surface and cell envelope fractions were routed through ESX-5. Additionally, saturated transposon-directed insertion-site sequencing (TraDIS) was applied to both wild-type *M*. *marinum* cells and cells expressing *mspA* to identify genes that are not essential anymore in the presence of MspA. This analysis confirmed the importance of *esx-5*, but we could not identify essential ESX-5 substrates, indicating that multiple of these substrates are together responsible for the essentiality. Finally, examination of phenotypes on defined carbon sources revealed that an *esx-5* mutant is strongly impaired in the uptake and utilization of hydrophobic carbon sources. Based on these data, we propose a model in which the ESX-5 system is responsible for the transport of cell envelope proteins that are required for nutrient uptake. These proteins might in this way compensate for the lack of MspA-like porins in slow-growing mycobacteria.

## Author Summary

*Mycobacteria* have a thick protective outer membrane that helps them to withstand adverse conditions both outside and within the host. However, in order to cause disease, the bacterium also needs to secrete proteins across this outer membrane. To achieve this, mycobacteria possess so-called type VII secretion systems. One of these systems, the ESX-5 secretion system, is only present in the group of slow-growing mycobacteria, which contains most pathogenic species. In this study, we show that the ESX-5 system is essential for growth of mycobacteria. We found that when we generated a ‘leaky’ outer membrane, by interfering in the construction of the outer membrane, or by introducing an outer membrane porin, the ESX-5 system was no longer essential for growth. We additionally show that ESX-5 mediates uptake of fatty acids, which suggests that ESX-5 substrates can form specific transport systems or pores in the outer membrane required for the uptake of crucial nutrients. Understanding the role of ESX-5 in outer membrane permeability helps us to understand a fundamental difference between fast-growing and slow-growing mycobacteria. Since most pathogenic mycobacteria are slow-growing this helps us to understand the mycobacterial requirements for pathogenesis in more detail.

## Introduction

*Mycobacterium tuberculosis* is one of the most important bacterial pathogens; this pathogen has infected almost thirty percent of the world population and is responsible for 1.4 million deaths annually [1]. A key characteristic that makes *M*. *tuberculosis* such a successful pathogen is the composition of its cell envelope. Mycobacteria and other families of the Actinobacteria belonging to the suborder Corynebacteriales [2] have a specialized outer membrane consisting of long chain (C_50_-C_90_) α-alkyl β-hydroxy fatty acids, known as mycolic acids. These mycolic acids are covalently linked to the arabinogalactan, which is in turn connected to the peptidoglycan matrix that is located in a periplasmic-like space. Mycolic acids, together with a wide range of other (glyco)lipids, such as lipooligosaccharides (LOSs) [3,4], phthiocerol dimycocerosates (PDIMs) [5,6] and trehalose mycolates [7], form a hydrophobic barrier that is organized as an outer membrane which is microscopically similar to that of Gram-negative bacteria [8,9]. This mycobacterial outer membrane functions as a highly efficient permeability barrier and plays a major role in the persistent nature of mycobacterial infections. It allows the bacteria to survive inside host phagosomes due to an increased resistance to bactericidal host factors such as oxidative radicals and antimicrobial peptides [10] and is also one of the main reasons for the antibiotic tolerance of mycobacteria [11].

In order to secrete virulence factors and other proteins over their specific cell envelope, mycobacteria have evolved the specialized type VII secretion (T7S) or ESX systems. T7S systems are found throughout the phylum of Actinobacteria and more distantly related systems are also present in Firmicutes. Thus far most information on T7S functioning and role in virulence has come from studying diverse mycobacterial species such as *M*. *tuberculosis*, the vaccine strain *Mycobacterium bovis* BCG, the closely related fish pathogen *Mycobacterium marinum* and the evolutionary more distant and avirulent *Mycobacterium smegmatis* [12–14]. Pathogenic mycobacteria contain up to five different T7S systems named ESX-1 to ESX-5 [15], which have probably evolved through duplication events [16]. These *esx*-loci are composed of several conserved genes, of which five encode for membrane components. Four of these membrane proteins, called EccB,C,D and E together form a large membrane complex, needed for protein transport [17]. One of these proteins, EccC, is a putative FtsK/SpoIII-like ATPase with three nucleotide binding domains (NBDs) and is hypothesized to play a central role in substrate recognition [18]. The fifth conserved membrane component of ESX systems is MycP, which is a subtilisin-like protease that is essential for secretion, although it is not part of the membrane complex.

ESX-1 was the first ESX system that was discovered [19] and is involved in the secretion of the important virulence factors EsxA (ESAT-6) and EsxB (CFP-10) as well as several other substrates [20]. Virulence of ESX-1 mutants is severely attenuated in macrophage cell lines and *in vivo* infection models, partially because they seem to be unable to escape the phagolysosome of macrophages [21,22]. Deletion of a large part of the ESX-1 genetic locus is also the major cause of the attenuation of the vaccine strain *M*. *bovis* BCG [19]. The ESX-3 system seems to have a very different function, as it is involved in iron and zinc uptake [23,24] and is therefore essential for growth of *M*. *tuberculosis*. ESX-5 is an intriguing system because it is only present in the slow-growing species of mycobacteria, which include most pathogenic species. This system is responsible for the secretion of many members of the large PE and PPE protein families in *M*. *marinum* [25]. A major group of these ESX-5 substrates are the glycine-rich and repetitive PE_PGRS proteins, which have been postulated to be involved in virulence [26,27] and immune evasion [28]. Nevertheless, the precise role of ESX-5 and its substrates has not been elucidated yet.

To understand the role of ESX-5 in virulence and the mechanism of secretion by this system, we aimed to select a wide range of ESX-5 mutants in *M*. *marinum*. However, in previous screening assays we only identified transposon insertions in genes encoding the cytosolic chaperone EspG_5_ and the cytosolic EccA_5_ [25,29,30], but not in any of the membrane components that make up the actual membrane transport machinery [17]. The inability to find mutations in these genes suggests that these mutations are in fact lethal for the cell. Indeed, Di Luca and colleagues recently showed that one of the genes encoding an ESX-5 membrane component is essential in a strain of *M*. *tuberculosis* (H37Rv) [31,32]. However, this effect was not observed in another *M*. *tuberculosis* strain (CDC1551) [17]. In this study, we show that ESX-5 membrane components are indeed essential for *in vitro* growth of *M*. *marinum* and *M*. *bovis* BCG. Strikingly, this essentiality can be circumvented by permeabilization of the outer membrane. Finally, we provide evidence that ESX-5 is involved in the uptake of nutrients and propose a model linking ESX-5 substrates to nutrient uptake and essentiality.

## Results

### *eccC*_*5*_ and *mycP*_*5*_ are essential for growth of *M*. *marinum*

To investigate the role of individual *esx-5* genes in secretion and virulence of *M*. *marinum*, these genes were deleted by allelic exchange using a specialized transducing mycobacteriophage [33]. We initially focused on *eccC*_*5*_ and *mycP*_*5*_, as both these genes are coding for highly conserved membrane components of the ESX-5 secretion system [17], but EccC_5_ is part of the membrane complex, whereas MycP_5_ is not and must have a separate function in ESX secretion. All attempts to delete these two genes in *M*. *marinum* were unsuccessful. To test whether this was due to the essentiality of these genes, we first introduced an integrative plasmid containing a kanamycin resistance cassette and the *eccB*_*5*_-*eccC*_*5*_ operon (pMV-*eccBC*_*5*_ *-kan*) [32], or *mycP*_*5*_ (pMV-*mycP*_*5*_ *-kan*). Using these merodiploid strains, the endogenous *eccC*_*5*_ gene (Table 1, upper two rows) or *mycP*_*5*_ gene, respectively, could readily be deleted, indicating that these genes are indeed essential.

**Table 1:** Essentiality of *eccC*_*5*_ and analysis of functional domains.

*input DNA*	*merodiploid* (WT*+ pMV-kan-eccBC*_*5*_)	*delinquent* (Δ*eccC*_*5*_ *+ pMV-kan-eccBC*_*5*_)
empty vector	+	-
*eccBC*_*5*_-WT	+	+
*eccB*_*5*_ P145 stop	+	+
*eccC*_*5*_ V57 stop	+	-
*eccC*_*5*_ R1365stop	+	-
*eccC*_*5*_ K506A (NBD1)	-*	-
*eccC*_*5*_ K879A (NBD2)	+/-* ^$^	+/-^$^
*eccC*_*5*_ R1181A (NBD3)	+/-* ^$^	+/-^$^
*eccC*_*5*_ R1181K (NBD3)	+	+

* The *eccC*_*5*_ NBD mutants appear to have a dominant negative effect on the functioning of endogenous EccC_5_.

^$^ Colonies showed a strong growth defect, i.e. colonies were visible only after 17 days, compared to 10 days for the wild-type strain.

Replacement of pMV-*eccBC*_*5*_ by the input DNA was scored. Input DNA consisted of the pMV-361-*hyg* plasmid containing the indicated constructs. “+” indicates that more than 100 colonies were detected after electroporation with the indicated vector. “–” indicates between 0–20 colonies were found after electroporation. These latter colonies were shown by PCR to still contain the original vector, indicating illegitimate recombination or spontaneous antibiotic resistance. Results are representative data of three independent experiments.

To confirm the essentiality of EccC_5_ and MycP_5_, a switching procedure of the complementation vectors was used in which we replaced the original construct with either a complementation plasmid (pMV-*eccBC*_*5*_ *-hyg* or pMV-*mycP*_*5*_ *-hyg*), or an empty version of this integrative plasmid (pMV361-*hyg*). These plasmids integrate at the same site, but contain a hygromycin resistance cassette instead of the kanamycin cassette [34]. Introduction of the complementation vectors resulted in successful switching to hygromycin-resistant colonies. PCR analysis confirmed proper exchange of plasmids in these colonies. In contrast, introduction of the empty vector produced no colonies that were hygromycin-resistant and kanamycin-sensitive. The few colonies that appeared after hygromycin selection were all kanamycin resistant, indicative of illegitimate recombination. PCR analysis confirmed the presence of both plasmids in these bacteria. Finally, we tested whether possible polar effects on *eccB*_*5*_ expression, which could be caused by the endogenous *eccC*_*5*_ deletion were responsible for the observed phenotype. The effect of a stop codon in either the *eccB*_*5*_ or *eccC*_*5*_ gene in the integrative plasmid during the switching procedure, was scored. Successful introduction of plasmids containing a stop codon in *eccC*_*5*_, either in the middle or at the end of the gene, was not possible, whereas introduction of an integrative plasmid with a stop codon in the beginning of *eccB*_*5*_ resulted in a high number of switch mutants (Table 1, rows 3–5). This experiment confirms that deletion of *eccC*_*5*_ has no negative polar effects on *eccB*_*5*_. Together, these data strongly suggest that the two ESX-5 genes, *eccC*_*5*_ and *mycP*_*5*_, are required for *in vitro* growth of *M*. *marinum*.

### MycP_5_ is essential for growth of *M*. *bovis* BCG

**Fig 1:**
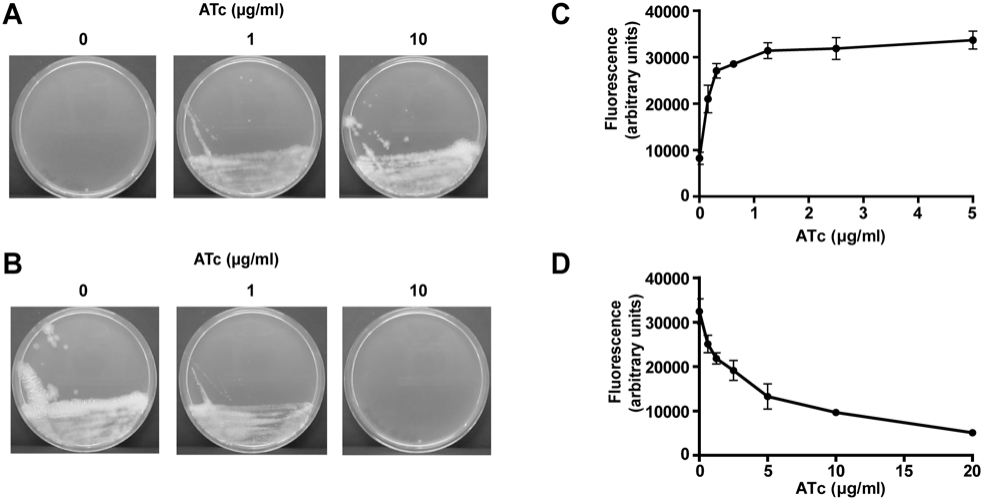
Expression of MycP5 is essential for growth of *M*. *bovis* BCG. A, B) The BCG-Pasteur c-*mycP5*-tet-on (A) and c-*mycP5*-tet-off (B) mutants were grown for 21 days on Middlebrook 7H10 agar plates containing the indicated ATc concentrations. Full growth of c-*mycP5*-tet-on was only observed at 10 μg/ml ATc, whereas this concentration of ATc did not completely abolish colony growth of c-*mycP5*-tet-off. C, D) Resazurin reduction is dependent on ATc-induced expression/repression of *mycP5*. Cells of the BCG-Pasteur c-*mycP5*-tet-on (C), or c-*mycP5-*tet-off (D) mutants were grown as liquid cultures in 96-well microtiter plates for 6 days at 37°C at the indicated ATc concentrations, after which 10% Alamar Blue was added and fluorescence (585 nm) was measured after 16 h incubation to determine metabolic activity as a correlate of growth. Values are means of triplicates; error bars represent the standard deviation.

To determine whether ESX-5 essentiality is also observed for the live vaccine strain *M*. *bovis* BCG, we created both a conditional *mycP*_*5*_ expression strain and a conditional *mycP*_*5*_ depletion strain by introducing a heterologous promoter including a multicopy *tetO* cassette immediately upstream of the *mycP*_*5*_ gene of *M*. *bovis* BCG Pasteur. This regulatable *mycP*_*5*_ gene was tested both in combination with a Tet repressor (TetR) protein exhibiting high-binding affinity to the *tetO* sites in absence of the inducer anhydrotetracycline (ATc; for establishing a *mycP*_*5*_ tet-on system) or in combination with a mutated TetR protein with reversed binding affinity to *tetO* sites upon binding of ATc (for establishing a *mycP*_*5*_ tet-off system) [35]. The c-*mycP*_*5*_-tet-on strain was unable to grow on 7H10 plates without ATc (Fig 1A), while full growth was only observed at a concentration of 10 μg/ml ATc. Inversely, when c-*mycP*_*5*_ *-*tet-off was grown on 7H10 plates supplemented with 10 μg/ml ATc, i.e. the *mycP*_*5*_ depleting condition, colony growth was suppressed (Fig 1B), although some colonies were still visible, possibly due to revertants of the tet-off system.

In addition, growth of c-*mycP5*-tet-on and c-*mycP5*-tet-off in liquid culture was tested using a resazurin reduction assay as a correlate of growth. By growing an inoculum of bacteria in the presence of varying concentration of ATc, we showed that growth of c-*mycP5*-tet-on correlated with ATc-dependent expression of *mycP*_*5*_ (Fig 1C). In contrast, growth of c-*mycP5*-tet-off was clearly inhibited in an ATc dependent matter (Fig 1D. Together these results show essentiality of *mycP*_*5*_ expression, and/or the downstream genes *eccE*_*5*_ and *eccA*_*5*_, for *in vitro* growth and metabolic activity of *M*. *bovis* BCG.

### Increasing the permeability of the mycobacterial outer membrane rescues the essentiality of ESX-5

We reasoned that the ESX-5 system could be essential for slow-growing mycobacteria due to toxic accumulation of an ESX-5 dependent substrate(s). In order to identify this putative toxic substrate *M*. *marinum* transposon mutants were selected that tolerate the deletion of *eccC*_*5*_. First, we generated a transposon library in the *M*. *marinum eccC*_*5*_ mutant, complemented with the integrative vector pMV-*eccBC*_*5*_ *-kan*. Subsequently, a switching procedure was performed with an empty vector. Two transposon mutants were identified, in which proper switching of the two plasmids had occurred. Interestingly, the transposons in these mutants were located in the genes *mas* and *ppsD*, both of which are reported to be involved in the biosynthesis of PDIMs and phenolic glycolipids (PGLs) [3,5,6]. Biochemical analysis showed that the *mas*::*tn* mutant indeed lacked PDIMs in its lipid extracts (S1 Fig). To verify that the transposon insertions in these genes were indeed responsible for rescuing essentiality of *eccC*_*5*_, complementation experiments were carried out using the switching approach described above. We generated various complementation plasmids carrying only the *mas* gene, or *mas* together with either the wild-type *eccBC*_*5*_ operon or the *eccBC*_*5*_ operon with a stop codon in *eccC*_*5*_. These plasmids were introduced in the *mas* or *ppsD* transposon mutant containing the *eccC*_*5*_ deletion, or in the complemented *eccC*_*5*_ mutant with an intact *mas* gene. Subsequently, we scored legitimate switching events in these mutants (Table 2). Complementation of the *mas* mutation in the *eccC*_*5*_ deletion background was only tolerated when the introduced plasmid simultaneously complemented the *eccC*_*5*_ deletion. This confirms that the absence of Mas is responsible for rescuing the essentiality of EccC_5_ for growth.

**Table 2:** Complementation of *mas*::*tn* in the Δ*eccC*_*5*_ mutant by replacement of the integrated pMV vector.

*input DNA*	*M*. *marinum wt*	Δ*eccC*_*5*_ *+ pMV-eccBC*_*5*_	Δ*eccC*_*5*_ *+ pMV361+ ppsD*::*tn*	Δ*eccC*_*5*_ *+ pMV361+ mas*::*tn*
empty vector	+	-	+	+
*eccBC*_*5*_	+	+	+	+
*mas*	+	-	+	-
*eccBC*_*5*_ *stop-mas*	+	-	+	-
*eccBC*_*5*_ *-mas*	+	+	+	+

Indicated strains were electroporated with the input DNA shown on the left. Input DNA consisted of the pMV-361-*hyg* plasmid containing the indicated constructs. Valid insertion of input DNA was scored as + or-, similarly as described for Table 1. Results are representative data of three independent experiments.

**Fig 2:**
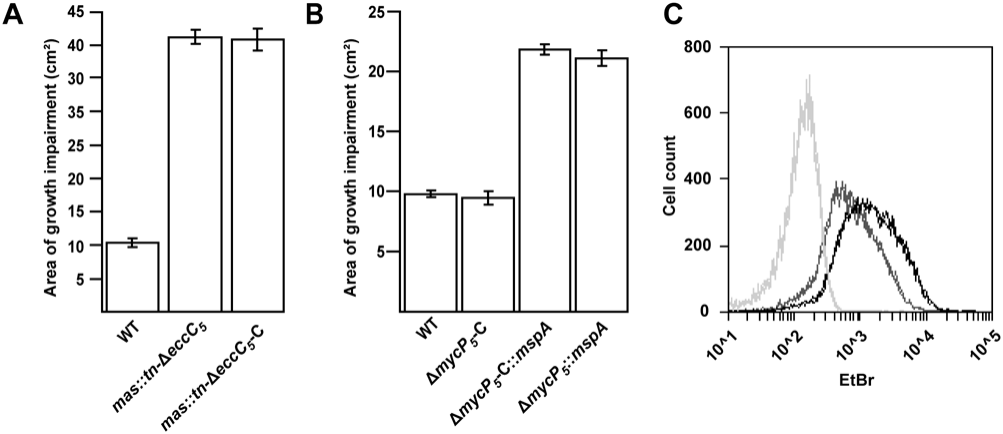
*mas* mutation or introduction of *mspA* lead to increased outer membrane permeability. A, B) Sensitivity to a combination of ampicillin and clavulanic acid of different *M*. *marinum* strains was measured by performing a disc diffusion assay on the indicated strains and measuring the surface of the growth inhibition zone. The *mas* transposon (*mas*::*tn)* mutants exhibit increased sensitivity, independent of the presence of an intact copy of *eccC5* (A). Similarly, introduction of *pSMT3*::*mspA* also leads to an increase in sensitivity independent on the presence of *mycP5* (B). Values are the means of triplicates; error bars indicate the standard deviation. C) Uptake of EtBr, measured by flow cytometric analysis. *M*. *marinum* wild-type (dark grey) and *M*. *marinum*::*mspA* (black) were incubated with 20 μM EtBr for 60 min and 20.000 events were analyzed for their fluorescence intensity at 585/540 nm. Light-grey lines indicate unstained samples. All measurements are depicted in duplicates and are representatives of three independent experiments. *ΔeccC5*-C and *ΔmycP5*-C refer to the complementation strains of the *M*. *marinum ΔeccC5* or the *ΔmycP5* mutants complemented with pMV::*eccBC5* or pMV::*mycP5* respectively.

Although the mutations affecting PDIM/PGL biosynthesis were clearly associated with ESX-5 essentiality they did not seem to be linked to putative lethal substrates. Therefore, we had to reevaluate our hypothesis. Interestingly, mutants in the PDIM/PGL biosynthesis locus of *M*. *marinum* are known to be more sensitive towards various antibiotics [6], indicating that the integrity of the cell envelope is affected. To test whether this was also true for the *M*. *marinum mas*::*tn-ΔeccC*_*5*_ strain we tested its resistance against a combination of ampicillin and the beta-lactamase inhibitor clavulanic acid via disc diffusion. Clavulanic acid is included in this assay because *M*. *marinum* contains a chromosomally encoded beta-lactamase. Since both ampicillin and clavulanic acid have their target in the periplasm, growth impairment is indicative for a compromised outer membrane. The *mas*::*tn-ΔeccC*_*5*_ mutant indeed showed increased ampicillin/clavulanic acid sensitivity as compared to wild-type *M*. *marinum*. This phenotype was not affected by complementation of *eccC*_*5*_ (Fig 2A) and was therefore probably due to the lack of PDIM production.

Bacterial outer membranes function as permeability barriers and therefore so-called porin proteins are usually present to allow passive diffusion of small hydrophilic molecules [36–38]. The most-studied mycobacterial porin is MspA, which was identified in the fast-growing, nonpathogenic species *Mycobacterium smegmatis*. Orthologues of this porin can be found in other fast-growing mycobacteria, but are generally not found or produced in slow-growing mycobacterial species, such as *M*. *tuberculosis* and *M*. *marinum*. We therefore hypothesized that introduction of MspA in *M*. *marinum* would lead to a more permeable outer membrane and could therefore possibly also rescue the essentiality of ESX-5. To this end, we introduced an *mspA*-expressing plasmid in the *M*. *marinum* Δ*eccC*_*5*_ and Δ*mycP*_*5*_ complemented strains. Introduction of this plasmid indeed increased antibiotic sensitivity in the Δ*mycP*_*5*_ complemented strain (Fig 2B) and also resulted in increased uptake of ethidium bromide in wild-type *M*. *marinum* (Fig 2C), which shows that MspA is functionally expressed in these strains [6,39]. Subsequently, the switching procedure with empty vector and *eccC*_*5*_ or *mycP*_*5*_-expressing plasmids was conducted as before. Strikingly, both the empty and complementation vectors resulted in successful switching, showing that introduction of MspA indeed alleviates the requirement of *eccC*_*5*_ and *mycP*_*5*_ for growth. The permeability of *mspA*-expressing strains was not affected by the presence of an intact *mycP*_*5*_ (Fig 2B). Tolerance for ESX-5 mutations was not due to spontaneous mutations in PDIM biosynthesis genes, as PDIM levels of these mutants were comparable to wild-type levels (S2 Fig). These findings confirm that increasing the permeability of the mycobacterial outer membrane rescues the essentiality of ESX-5 in *M*. *marinum*.

**Fig 3:**
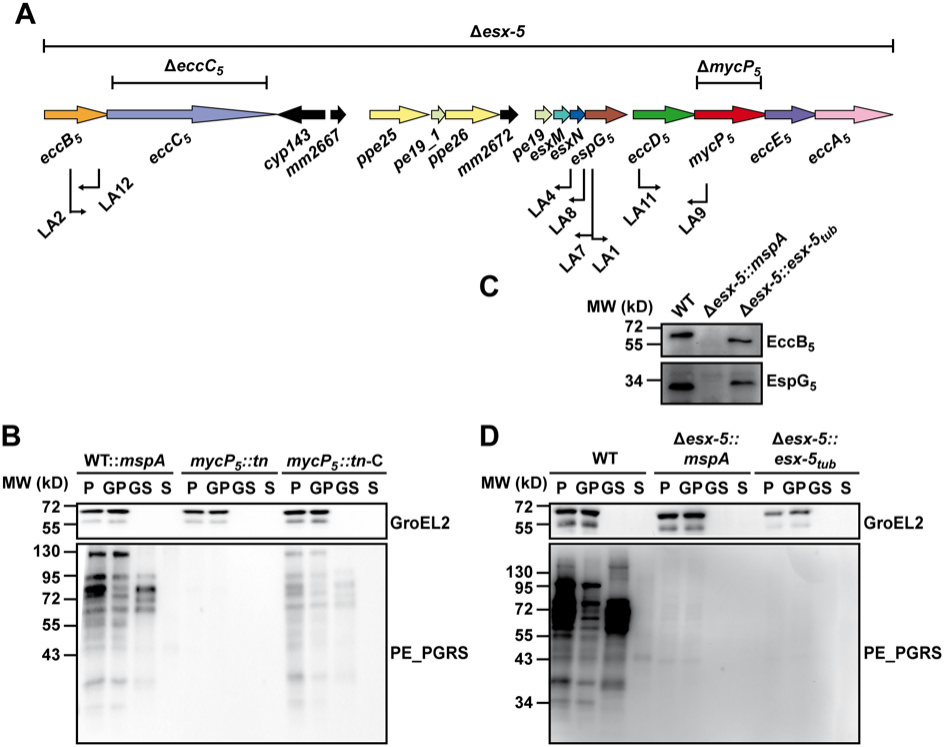
Secretion analysis of ESX-5 mutant strains. A) A schematic representation of the ESX-5 region of *M*. *marinum* with the different ESX-5 mutations used in this study. Bars above the gene cluster indicate regions deleted by targeted knock-out mutagenesis. Arrows below indicate position and orientation of transposons (named LA1 to LA12) in mutants of the parental strain *M*. *marinum*::*mspA* defective in ESX-5 dependent secretion. B) Secretion analysis of *M*.*marinum*::*mspA (*WT::*mspA)*, a *mycP5* transposon mutant (*mycP5*::*tn*, corresponding to LA9 in (A)) and the complemented version of this strain (*mycP5*::*tn*-C). Secreted proteins (S) were separated from bacterial cells (P) by centrifugation. In addition, surface-associated proteins were enriched from the bacterial cells by extraction with 0.5% Genapol X-080 (GS) and separated from non-solubilized proteins (GP) by centrifugation. All fractions were analyzed for the presence of PE_PGRS proteins by immunoblotting. GroEL2 staining was used as a loading and lysis control. C) Expression of EccB_5_ and EspG_5_ was analyzed by immunoblotting of total cell lysates of wild-type *M*. *marinum* (WT), the *Δesx-5*::*mspA* mutant and the complemented *Δesx-5*::*esx-5tub* strain. D) The same strains as under (C) were analyzed for their ability to express and secrete PE_PGRS proteins following the same procedure as under (B).

We previously failed to isolate transposon mutants in any of the genes encoding ESX-5 membrane components by screening transposon mutant libraries for secretion defects [25,29]. To determine whether we could now isolate such transposon mutants by introduction of MspA, we repeated our original screens using a transposon library created in *M*. *marinum* expressing *mspA*. This transposon library of ~10.000 mutants was screened for the secretion of the ESX-5 dependent PE_PGRS proteins, using the previously described double filter assay [29]. In total, eight transposon mutants were identified that showed completely abolished PE_PGRS secretion. All eight secretion mutants had transposon insertions in the ESX-5 region (Fig 3A), four of which were affected in genes encoding the membrane components *eccB*_*5*_, *eccD*_*5*_ and *mycP*_*5*_. Secretion analysis confirmed that these ESX-5 transposon mutants showed strongly reduced expression and secretion of PE_PGRS proteins and lacked expression of the mutated components (Figs 3B and S3). To show reversibility of the phenotype, the MycP_5_ transposon mutant (LA9) was complemented (Fig 3B). In conclusion, the introduction of MspA allowed transposon insertions in the ESX-5 locus and only mutants within the ESX-5 gene cluster showed a complete lack of PE_PGRS secretion, underscoring the importance of this locus in this process.

### The role of the nucleotide binding domains of EccC_5_ in essentiality and secretion

**Fig 4:**
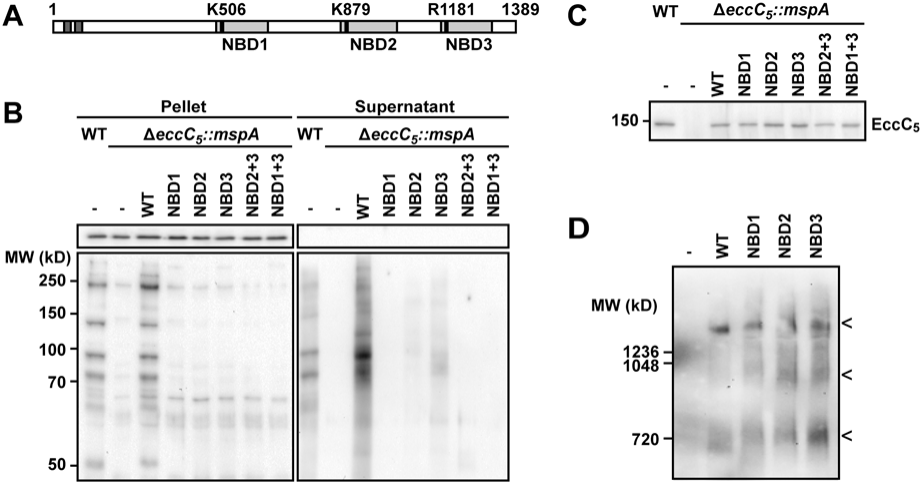
Role of NBDs domains of EccC5 in ESX-5 dependent secretion and membrane complex assembly. A) Predicted transmembrane domains (dark grey), and NBD (light grey) of EccC_5_ are indicated. The positions of relevant residues are depicted with a black bar. The numbers represent the position in amino acids. B) Secretion of PE_PGRS proteins in the different EccC_5_ mutant strains was analyzed by immunoblot of supernatants and cell pellets of wild-type (WT) *M*. *marinum* and the *eccC5* deletion strain *(*Δ*eccC5*) complemented with various *eccC5* mutated genes. GroEL2 staining was used as a control for lysis and equal loading. C) Immunoblot analysis of EccC_5_ expression in isolated membranes of indicated strains. D) Blue native PAGE and immunoblot analysis using an anti-EccD_5_ antibody of the ESX-5 membrane of *M*. *marinum* Δ*eccC5*::*mspA*, complemented either with an empty vector (-) or with various *eccC5* mutated genes. For all samples that contained EccC_5_ variants the characteristic pattern of ESX-5 membrane complexes was observed [28], consisting of the largest ~1.5 MDa complex and two additional smaller subcomplexes (indicated by the three arrowheads).

Because ESX-5 essentiality can be rescued by introduction of MspA, we hypothesized that one or more of the ESX-5 substrates could be responsible for the essentiality. However, it is also possible that the presence of the ESX-5 membrane complex itself is responsible for this phenomenon. We reasoned that we could distinguish between these possibilities by further dissecting the role of the membrane component EccC_5_. EccC_5_, together with EccB_5_, EccD_5_ and EccE_5_, forms a large ~1.5 MDa complex in the mycobacterial cell envelope [17] that likely constitutes the membrane channel through which substrates are transported. In addition, EccC is predicted to be an ATPase with three nucleotide binding domains (NBDs). These NBDs usually contain a characteristic lysine residue in the Walker A motif, essential for ATP binding. However, the third NBD of EccC_5_ has an arginine at this position, which is a conserved feature for NBD3 of ESX-5 systems (Fig 4A). Based on analogy to other ATPases involved in secretion [40,41], the NBDs of EccC play either a role in substrate transport or in the assembly of the membrane complex. We reasoned that by mutating these domains we could determine which function (i.e. in secretion or complex formation) is essential for bacterial growth.

EccC_5_ variants with point mutations in the 3 NBD domains were introduced in the *eccC*_*5*_ knock-out strain using the switching procedure outlined previously. In these EccC_5_ variants the conserved lysine residue in NBD1 or NBD2 was replaced by an alanine, whereas the arginine residue in NBD3 was replaced by either by an alanine (R1181A) residue or the preferred lysine residue (R1181K) (Table 1, rows 6–9). While valid plasmid exchange was observed for the NBD2, NBD3-R1181K and NBD3-R1181A mutants, no valid exchange was observed for the NBD1 mutant, indicating that this first NBD is crucial for EccC_5_ functioning. Although the NBD2 and NBD3 R1181A mutations were tolerated, these mutants showed a significant growth defect on plate. In contrast, NBD3-R1181K did not show any growth inhibition. Strikingly, when the same plasmids were introduced in wild-type *M*. *marinum*, the same growth inhibition phenotype was observed, suggesting that these EccC_5_ NBD mutants have a dominant negative effect on the functioning of endogenous EccC_5_.

Next, we studied the effect of the mutations in EccC_5_ on PE_PGRS secretion via the ESX-5 system. For this, we carried out the plasmid switching procedure similarly as above, but now using the *eccC*_*5*_ knock-out strain that additionally contained the MspA-expressing vector (Fig 4B). The NBD1 mutation completely abolished the presence and secretion of PE_PGRS proteins, while NBD2 and NBD3-R1181A mutations strongly reduced expression and secretion of these ESX-5 substrates. These data show a strong correlation between the level of secretion of ESX-5 substrates and the essentiality of EccC_5_ for growth. Importantly, the lack of PE_PGRS secretion was not due to instability of the various EccC_5_ mutants, as was shown by western blot analysis of isolated cell envelope fractions (Fig 4C). Furthermore, Blue Native PAGE analysis of detergent-solubilized membrane proteins showed that formation of the EccBCDE_5_ membrane complex was also not affected by these NBD mutations (Fig 4D). We conclude that none of the three NBDs of EccC_5_ is involved in assembly of the ESX-5 membrane complex, suggesting that they are dedicated to energize the transport of substrates over the cell envelope. In turn, this indicates that proteins transported by ESX-5 and not the system itself are responsible for the essentiality of ESX-5.

### Deletion of *M*. *marinum* ESX-5 and complementation with the ESX-5 region of *M*. *tuberculosis*

The identification of a strategy to generate viable ESX-5 deletion strains also allowed us to determine the secretome of an *esx-5* null-mutant. A targeted knock-out of the complete ESX-5 gene cluster of *M*. *marinum*, spanning the genes *eccB*_*5*_ to *eccA*_*5*_ (Fig 3A) was created in the presence of MspA (*Δesx-5*::*mspA*). In addition, we introduced an integrative plasmid containing the *esx-5* locus of *M*. *tuberculosis* (pMV::*esx-5*_*tub*_) [17] in wild-type *M*. *marinum*, after which the endogenous ESX-5 region was deleted as described above (*Δesx-5*::*esx-5*_*tub*_). Interestingly, this approach was possible in the absence of MspA, although this strain showed slightly slower growth than wild-type bacteria. These data show that the ESX-5-region of *M*. *tuberculosis* can, at least partially, take over the essential role of the ESX-5 system of *M*. *marinum*.

In order to assess whether introduction of the *esx-5* locus of *M*. *tuberculosis* also leads to a fully functional complementation, we analyzed the expression and secretion of PE_PGRS proteins (Fig 3D) and expression of EspG_5_ [42] and EccB_5_ [17] (Fig 3C). While expression of both EspG_5_ and EccB_5_ was indeed restored, expression and secretion of PE_PGRS proteins were not (Fig 3D). This shows that the *M*. *tuberculosis* ESX-5 region is not able to fully complement its *M*. *marinum* orthologues.

Next, the secretome of these newly constructed strains was analyzed by mass spectrometry. Since we have previously shown that the majority of ESX substrates in *M*. *marinum* remains attached to the cell surface, rather than being secreted into the growth medium [43], we focused on cell-surface proteins in this analysis. As a control we also determined the proteome of the cell envelope fraction obtained after cell disruption. Cell surface proteins can be released by incubating intact bacteria with the detergent Genapol X-080 [44]. Because this procedure also results in the extraction of low amounts of cell envelope proteins [43], we compared these fractions with bona fide cell envelope fractions and selected for Genapol-enriched proteins. *M*. *marinum* wild-type, *M*. *marinum*::*mspA*, *Δesx-5*::*mspA* and *Δesx-5*::*esx-5*_*tub*_ strains were grown in liquid culture, after which cell envelope fractions were isolated using cell disruption and centrifugation or surface proteins were isolated using Genapol-X080 extraction. Protein samples from two independent experiments were analyzed by LC-MS/MS and spectral counts were used to measure relative abundance of proteins across the different strains and fractions (S1 File). First, cell surface enriched and cell envelope fractions of the wild-type strain were normalized and compared. Proteins that had four-time higher relative abundance in the cell surface enriched fractions as compared to the cell envelope fraction were defined as probable cell surface proteins. This created a list of 114 proteins that contained many known surface-associated proteins and a number of lipoproteins (S1 File). This subset of putative surface proteins was compared between the strains *M*. *marinum*::*mspA* and *Δesx-5*::*mspA*, resulting in a list of 30 proteins that were at least five-fold less abundant in the *esx-5*-null mutant (Table 3). Please note that half of these proteins are completely absent in the *esx-5* mutant. As expected, the majority (24) of these putative ESX-5 substrates were PE and PPE proteins, of which 17 were PE_PGRS proteins and 7 PPE proteins. Besides the PE/PPE proteins that were detected, six proteins with no apparent link to ESX-5 are also in this list. Five of these six proteins are annotated as secreted or surface associated proteins and contain canonical N-terminal signal sequences, which makes it most-likely they are exported by the Sec machinery [45]. Together, these results confirm and extend our previous results from mutants in individual ESX-5 genes [25], and confirm that ESX-5 is the major secretion pathway of PE and PPE proteins.

**Table 3:** ESX-5-dependent surface proteins of *M*. *marinum*.

		MS/MS Normalized spectral counts*			Fold Change
Identified protein	Protein description	*M*.*marinum*::*mspA*	Δ*esx-5*::*mspA*	Δ*esx-5*::*esx-5*_*tub*_	wild-type/ Δ*esx-5*::*mspA*
MMAR_3105	PE-PGRS	87	0	0	∞
MMAR_4999	PE-PGRS	60	0	0	∞
MMAR_2459	PE-PGRS	54	0	2	∞
**MMAR_2656**	**PE-PGRS50**	**54**	**0**	**11**	**∞**
MMAR_3400	PE-PGRS	48	0	0	∞
iipB		46	0	2	∞
MMAR_1199	PE-PGRS	38	0	0	∞
MMAR_5135	PE-PGRS	37	0	0	∞
MMAR_1908	Membrane hydrolase	35	0	2	∞
MMAR_3316	PE-PGRS	32	0	0	∞
MMAR_2460	PE-PGRS	30	0	0	∞
MMAR_3728	PE-PGRS2	21	0	0	∞
MMAR_1130	PPE	21	0	0	∞
MMAR_4939	PE-PGRS	18	0	0	∞
**MMAR_4153**	**Conserved secreted**	**11**	**0**	**33**	**∞**
MMAR_1442	PE-PGRS	180	3	0	51
MMAR_2235	PE-PGRS PE16	118	3	0	34
MMAR_5013	PE-PGRS	43	3	0	14
MMAR_3665	PPE	94	10	27	10
MMAR_0382	PE-PGRS	65	7	2	9
**MMAR_0761**	**PPE10**	**62**	**7**	**51**	**9**
MMAR_4187	PPE	29	3	0	9
MMAR_1497	PPE28	57	7	0	9
MMAR_2973	PE-PGRS	82	10	0	8
MMAR_1402	PPE	153	19	4	8
MMAR_5047	PPE	50	6	0	8
MMAR_1547	PE-PGRS	25	3	2	8
**MMAR_2586**	**Conserved secreted**	**212**	**27**	**213**	**8**
**MMAR_3410**	**Pyruvate kinase**	**70**	**9**	**49**	**7**
**MMAR_0559**	**Conserved exported**	**43**	**6**	**11**	**7**

* Average normalized spectral counts from two biological replicates

Cell surface enriched and cell envelope proteins of *M*. *marinum*::*mspA and Δesx-5*::*mspA* strains were analyzed by LC-MS/MS. Proteins were classified as secreted proteins when the relative normalized abundance was four times higher in the cell-surface enriched fractions as compared to the cell envelope. Shown here are only proteins with at least a five-fold decrease in spectral counts in the Δ*esx-5*::*mspA* strain. Rows in bold indicate proteins with restored secretion in the presence of *esx-5*_*tub*_.

To quantify the extent of complementation by the *M*. *tuberculosis esx-5* locus we analyzed which ESX-5-dependent surface-associated proteins were five times more abundant in *Δesx-5*::*esx-5*_*tub*_ compared to *Δesx-5*::*mspA*. In concordance with the data obtained by immunoblot, the secretion of only a limited number of ESX-5 substrates was restored by the introduction of *esx-5*_*tub*_ (Table 3). Among these few substrates were two PE/PPE proteins, namely PPE10 (MMAR_0761) and PE_PGRS50 (MMAR_2656—PE_PGRS50). Previously, high density transposon mutagenesis analysis has shown that the genes encoding these proteins are not essential for *M*. *marinum* E11 [46], so either they are not the cause of the essentiality of ESX-5 or they are together responsible. The three other restored proteins are MMAR_4153, MMAR_3410 and MMAR_2586, all of which are not essential, do not have a T7S signal [47] and have not been linked to ESX-5 secretion previously.

Because our analysis of surface-associated proteins could not pinpoint essential ESX-5 substrates, we hypothesized from the observed link with outer membrane permeability that the essential ESX-5 substrate could be integrally inserted and therefore more stably associated with the outer membrane. Therefore, we also compared the cell envelope fractions of the same set of strains (S1 Table). As expected, the ESX-5 membrane components EccB_5_ to EccE_5_, and MycP_5_ were detected in large amounts in both wild-type *M*. *marinum* strains (i.e. with or without episomal *mspA*) as well as in *Δesx-5*::*esx-5*_*tub*_ but could not be detected in *Δesx-5*::*mspA*. This analysis furthermore showed that there were indeed two putative ESX-5 substrates detectable in the cell envelope fraction. MMAR_1129 is a PPE protein with similarities to *M*. *tuberculosis* PPE64, whereas MMAR_1442 is a PE_PGRS protein similar to the PE_PGRS27 in *M*. *tuberculosis*. Both these proteins are shown to be cell envelope localized in an ESX-5 dependent manner, but are not complemented by the *esx-5*-locus of *M*. *tuberculosis*. Again, the genes encoding these proteins are non-essential in *M*. *marinum* [46]. Interestingly, several proteins involved in lipid biosynthesis also seem affected by the ESX-5 mutation and are partially complemented in *Δesx-5*::e*sx-5*_*tub*_, suggesting that lack of a functional ESX-5 has implications for lipid metabolism. In summary, introduction of the ESX-5 region of *M*. *tuberculosis* leads to rescue of essentiality of the ESX-5 region in *M*. *marinum*, which shows that the essential role of ESX-5 is conserved. However, the ESX-5 region of *M*. *tuberculosis* is only marginally able to restore ESX-5-dependent secretion in *M*. *marinum*, suggesting not only that substrate recognition is (partially) species specific, but also that the major portion of the identified ESX-5 substrates do not cause the essentiality of the secretion system.

### Transposon directed insertion site sequencing

To directly identify ESX-5 substrates that cause the essentiality of ESX-5 we performed the genome-wide approach of transposon directed insertion site sequencing (TraDIS). This extensive technique allows the analysis of large libraries of random transposon insertion mutants and as such is able to identify which genes are essential under different conditions or in different genetic backgrounds [48,49]. We created >100,000 Mycomar transposon mutants of wild-type *M*. *marinum* and *M*. *marinum*::*mspA*, which number has been shown before to result in hitting 97% of all non-essential TA sites [46]. Subsequently, transposon insertion sites were determined by Illumina sequencing, the data were normalized and the number of transposon hits per gene was established. By comparing the two libraries we could identify genes that are specifically enriched (>3 fold) in bacteria expressing *mspA* (S2 File). As expected, the ESX-5 membrane components were found among the top hits (Table 4). For instance, transposon insertions in *eccD*_*5*_ were detected 1018-fold more in the *mspA*-expressing strain and similar patterns were seen for *mycP*_*5*_, (181x) *eccB*_*5*_, (62x), *eccE*_*5*_ (49x) and *eccC*_*5*_ (44x). Interestingly no ESX-5 substrates were identified among the top hits, although some genes encoding substrates were somewhat enriched. Insertions in *ppe1* (*mmar_0261*), *mmar_3290* (an *M*. *marinum* specific PE_PGRS) and *ppe59* (*mmar_4187*) were 3–4 fold more common when *mspA* was expressed. This result indicates that not a single ESX-5 substrate, but multiple proteins together are responsible for the essentiality of ESX-5.

**Table 4:** *M*. *marinum* genes with enriched numbers of transposon insertions in *M*. *marinum* supplemented with MspA.

				Insertion counts		
Gene ID	Gene description	H37Rv orthologue	Gene length (bp)	*M*. *marinum* wild-type	*M*. *marinum*::*mspA*	Normalized fold change
***mmar_2677***	***eccD*_*5*_**	***rv1795***	**1512**	**30**	**1463**	**1018**
*mmar_2221*	*ribC*	*rv1412*	624	2	104	283
***mmar_2678***	***mycP*_*5*_**	***rv1796***	**1743**	**36**	**840**	**181**
*mmar_5136*	DNA polymerase III	*Rv3664*	1218	3	126	140
*mmar_3746*	Lipase/esterase	*Rv2418*	756	8	590	127
*mmar_3941*	Phage protein	*-*	336	13	105	116
***mmar_2664***	***eccB*_*5*_**	***rv1782***	**1524**	**28**	**353**	**62**
***mmar_2679***	***eccE*_*5*_**	***rv1797***	**1212**	**12**	**124**	**49**
***mmar_2665***	***eccC*_*5*_**	***rv1783***	**4167**	**64**	**1443**	**44**
*mmar_1257*	*birA*	*Rv3279c*	831	4	122	30

Depicted are the number of transposon insertions detected by TraDIS in wild-type *M*. *marinum* or in a strain expressing MspA. The detected insertions are normalized for the total amount of reads per sample and genes are ranked based on the fold change between *M*.*marinum*::*mspA* divided by wild type *M*.*marinum*. The top ten hits are depicted. ESX-5 components are in bold.

This transposon mutagenesis approach did reveal other effects of the presence of MspA. Overall, insertions in genes involved in lipid metabolism seem to confer a growth advantage in *M*. *marinum*::*mspA*, even though many of these genes are not essential. For instance, mutations in *desA3* (*mmar_1315*), a stearoyl coenzyme A desaturase involved in the biosynthesis of oleic acid, are enriched 18-fold, reaching 0.12% of the amount of total insertions. A similar phenomenon can be observed for most genes of the *mce1* locus. Although insertions in these genes are not enriched dramatically, they can be observed over the complete gene cluster. (*yrbE1A* (4x), *mce1B* (3.7x), *mce1D* (3x), *mmar_0419* (4.7x) and *mmar_0421* (3.4x). Another affected gene known to play a role in lipid metabolism is *icl* (*mmar_1792*) encoding for the isocitrate lyase enzyme [50]. Together these data show that when *mspA* is expressed in *M*. *marinum* there is a shift in lipid metabolism, which could be due to higher availability of simple carbon sources, such as glucose and glycerol, by the presence of the hydrophilic pore. However, the most dramatic change is the reduced essentiality of *esx-5*. As this effect could almost exclusively be observed for *esx-5* genes and not for genes encoding ESX-5 substrates, the substrates that cause the essentiality of ESX-5 are likely redundant.

### The ESX-5 system is involved in nutrient uptake

Since essentiality of ESX-5 could be rescued by increasing outer membrane permeability, we hypothesized that ESX-5 substrates could be involved in the uptake of nutrients that are essential for growth. Because TraDIS analysis did indicate that this effect was probably due to multiple substrates, we set out to test whether we could find specific carbon sources that were not transported in the ESX-5 mutant. One limitation in this analysis is of course that these mutants always contain the large hydrophilic MspA pore.

**Fig 5:**
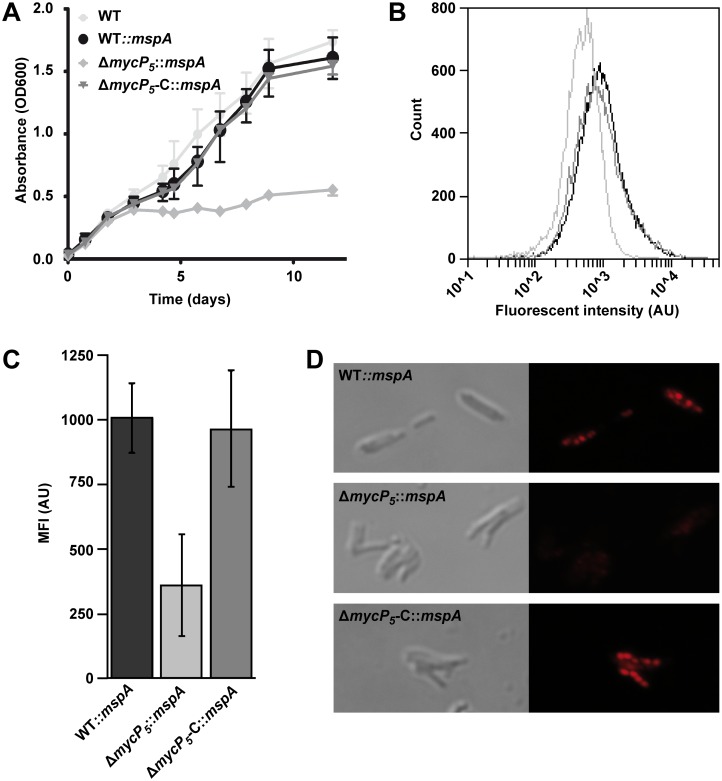
ESX-5 is involved in fatty acid uptake. A) Growth of indicated *M*. *marinum* strains on Tween-80 as a sole carbon source was assessed by measuring optical density at different time points. Depicted is the average of three biological replicates. Error bars indicate standard deviations. B) Uptake of a fluorescently labeled fatty acid after 72 hours of hypoxic growth was measured by FACS analysis. 20.000 events gated for similar size were acquired for WT::*mspA* (black), Δ*mycP5*::*mspA* (light grey) or Δ*mycP5-C*::*mspA* (dark grey). C) Quantification of FACS analysis. Mean fluorescent intensity of three experiments per strain was acquired by FACS. Background staining, quantified by adding the fluorescent fatty acid to an unstained culture one hour before washing the cells, was deducted from the measured values. Error bars indicate the standard deviations and One-way ANOVA showed a statistical difference between the samples of *p* = 0.010. D) Uptake of the fluorescently labeled fatty acid and formation of lipid bodies was confirmed by confocal microscopy.

To test whether the ESX-5 system is involved in the uptake of nutrients, *M*. *marinum*-Δ*mycP*_*5*_::*mspA* and several control strains were grown in a modified 7H9 medium supplemented with different single carbon sources [51]. The *mycP*_*5*_ mutant grew almost as fast as the control strains in the presence of small hydrophilic carbon sources such as glucose, glycerol or acetate (S4 Fig). This was expected, as these strains contained the hydrophilic pore-forming MspA protein [37,38]. However, when the strains were grown on medium with Tween-80 (Fig 5A) or Tween-40 (S4 Fig) as sole carbon source, only the *mycP*_*5*_ deletion strain showed strongly reduced growth. Mycobacteria are able to hydrolyze Tween and use the fatty acid components as carbon source. However, it is also known that free fatty acids can be toxic for mycobacteria. To discriminate between these two possibilities we added 0.2% glucose to the cultures after 8 days of growth on Tween-80. This resulted in normal outgrowth of the *mycP*_*5*_ mutant, indicating that the Tween-80 present in the medium did not specifically hamper growth of this strain, but that these cells were still viable and therefore probably starved (S4 Fig). This result indicates a role for the ESX-5 system in either the (extracellular) hydrolysis of Tween-80 or the uptake of released oleic acid. To examine whether ESX-5 secreted substrates are involved in the breakdown of Tween-80, co-culture experiments using wild-type *M*. *marinum* and *M*. *marinum*-Δ*mycP*_*5*_::*mspA* were performed. Growth of the *mycP*_*5*_ mutant strain was not rescued by the presence of wild-type bacteria (S4 Fig), indicating that factors secreted to the culture filtrate do not play a role in the observed growth defect. This was further confirmed by testing the role of the ESX-5 dependent lipase LipY in the ability of *M*. *marinum* to grow on Tween-80. LipY is the most active and abundant lipase secreted via ESX-5 [52] and therefore a prime candidate for hydrolyzing Tween-80. However, an *M*. *marinum lipY* deletion mutant grew to a similar extent as the wild-type strain on medium with Tween-80 as a sole carbon source (S4 Fig), showing that LipY is not responsible for the ESX-5 dependent growth on Tween-80.

Next, we investigated in a more direct manner whether ESX-5 mutants are able to import fatty acids. Under hypoxic conditions mycobacteria incorporate fatty acids as intracellular triacylglycerol (TAG) in so-called lipid bodies [53,54]. These lipid bodies can be visualized by adding BODIPY-labeled fluorescent fatty acids to hypoxic cultures. *M*. *marinum-*Δ*mycP*_*5*_::*mspA* and the corresponding control strains were grown in the presence of a fluorescently labeled fatty acid under hypoxic conditions. Uptake of this fatty acid was determined by measuring the fluorescence of the bacteria by FACS analysis (Fig 5B). The *mycP*_*5*_ *-*deletion strain showed significantly reduced fluorescent intensity as compared to the complemented or wild-type strains (Fig 5C). This effect was confirmed by confocal microscopy (Fig 5D); while the wild-type and the complemented *mycP*_*5*_ strain showed intracellular fluorescent lipid bodies, indicating that the fluorescent fatty acid was incorporated and stored in lipid bodies [53], no fluorescent lipid bodies were observed in the *mycP*_*5*_ deletion strain. These data together show that ESX-5 or one of it substrates is involved in the uptake of fatty acids. Together these data show that ESX-5 substrates are involved in the efficient utilization of fatty acids.

## Discussion

Previous results suggested that the ESX-5 system could be essential for growth of *M*. *marinum* [25,29,30]. Here, we show that the membrane components EccC_5_ and MycP_5_ are indeed essential for this species. In addition, silencing of the *mycP*_*5*_ gene in *M*. *bovis* BCG results in significantly reduced growth. This phenotype is similar to the phenotype of an *eccC*_*5*_ depletion strain of *M*. *tuberculosis* H37Rv [31]. Strikingly, we were able to obtain mutants in the ESX-5 core components when the outer membrane permeability was increased, either by mutating PDIM biosynthesis genes or by introduction of the *M*. *smegmatis* outer membrane porin MspA.

One hypothesis that would explain this observation is that a defect in ESX-5 dependent secretion could cause the accumulation of toxic molecules in the periplasm, which are able to exit the cell via the MspA porin or through the more permeabilized outer membrane. However, it is difficult to envision how the absence of the ESX-5 protein secretion system could cause the periplasmic/cytoplasmic accumulation of such molecules. We therefore favor and tested the alternative hypothesis that the ESX-5 system is involved in the influx of nutrients and/or other metabolites that are crucial for growth. In support of the second hypothesis we could show that ESX-5 mutations strongly affected the ability of *M*. *marinum* to grow on different polysorbate detergents (i.e. Tween-40 and Tween-80), suggesting that ESX-5 facilitates the usage of fatty acids as a carbon source. In addition, the ESX-5 mutant is also impaired in the intracellular accumulation of fluorescent fatty acids. Notably, the ESX-5-dependent growth on polysorbate-like detergents and the uptake of fatty acids were observed in the presence of the MspA porin. Fatty acid uptake therefore seems to be relatively specific for ESX-5. Pathogenic mycobacteria accumulate lipid bodies, presumably as energy storage, during the dormant stage of infection. Our data suggest that ESX-5 plays a central role in this crucial process in infection. Interestingly, earlier studies have shown that an *espG*_*5*_ mutant, which shows strongly diminished ESX-5 dependent secretion, is in fact hypervirulent in adult zebrafish [55]. It is possible that the inability of this mutant strain to take up fatty acids during infection prevents the bacterium to go into dormancy, resulting in outgrowth of the mutant in the host. Possibly, ESX-5 is also required for the utilization of other substrates. However, the uptake of hydrophilic substrates is more difficult to test, because we need the expression of the MspA porin when *esx-5* is deleted. Interestingly, also the ESX-3 secretion system of Mycobacteria is involved in nutrient import; this system is essential for the uptake of iron and zinc ions in *M*. *tuberculosis* [23]. Unfortunately, the exact mechanism for metal ion uptake via ESX-3 is not known. Our TraDIS analysis shows that essentiality of this system cannot be alleviated by the introduction of MspA.

**Fig 6:**
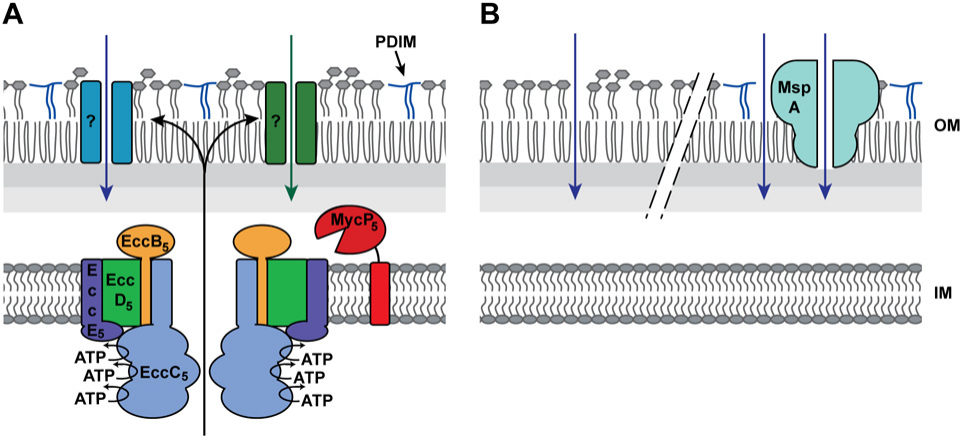
Model for the essentiality of ESX-5. A) Presented is the working hypothesis, in which the ESX-5 system is responsible for the insertion of several channel- or pore-forming protein (indicated by the question marks) that mediates the uptake of essential nutrients and/or other metabolites. B) The lack of PDIMs (left) or the presence of MspA-like porins (right) increases the permeability of the outer membrane, allowing the passive diffusion of the hypothesized essential nutrient(s), thereby circumventing the essentiality of ESX-5.

Introduction of MspA or defects in the biosynthesis of PDIM makes the outer membrane more permeable, which would allow passive diffusion of nutrients, thereby circumventing the requirement for the ESX-5-dependent nutrient uptake (Fig 6B). Interestingly, while a compromised outer membrane by the absence of PDIM likely enables a more efficient influx of hydrophobic solutes, MspA is a water-filled channel and allows predominantly diffusion of small hydrophilic molecules. This would suggest that ESX-5 is involved in the influx of both hydrophobic and hydrophilic molecules. However, the presence of MspA has been shown to also increase the sensitivity of *M*. *smegmatis* for hydrophobic antibiotics such as erythromycin, from which it was hypothesized that this porin might additionally affect the integrity of the outer membrane [39]. This perhaps also explains why we and others [10] observed an increase in uptake of ethidium bromide in the presence of MspA, while this molecule is theoretically too bulky to fit in the MspA channel. In addition, PDIM mutants have been shown, both in this study and by others [6,56], to be more sensitive to both hydrophobic and hydrophilic antimicrobials.

Which proteins are responsible for the ESX-5-dependent uptake of fatty acids and possibly also other nutrients? Our analysis of the NBDs of EccC_5_ indicates that it is not the presence of the ESX-5 membrane complex, but active secretion through this complex. The observed nutrient influx is therefore probably mediated by ESX-5 substrates that possibly form outer membrane porins or other types of outer membrane channels (Fig 6A). To identify these essential ESX-5 substrates, we analyzed the complete ESX-5 secretome of *M*. *marinum*. LC-MS/MS analysis showed that this mutant strain was deficient in secretion of all abundant PE and PPE proteins. The only PE/PPE proteins that have been shown previously to be independent on ESX-5 are the ESX-1 dependent PE35/PPE68_1 protein couple that is associated with the ESX-1 gene cluster [43,47]. These proteins were not detected in sufficient amounts in this study to draw any conclusions. Interestingly, while the ESX-5 region of *M*. *tuberculosis* was able to complement the *M*. *marinum* ESX-5 system for growth, only a small number of relatively low-abundant *M*. *marinum* ESX-5 substrates were secreted by the ESX-5 system of *M*. *tuberculosis*. Possibly, the recognition of ESX-5 substrates is relatively species specific or species-specific chaperones are required for efficient secretion. This is not surprising as the PE/PPE proteins show a high variation between species [57], and especially the more species-specific PE/PPE proteins are the ESX-5 substrates that are most highly expressed in *M*. *marinum* [25]. As *M*. *marinum Δesx-5*::*esx-5*_*tub*_ was viable, the partial complementation allowed us to generate a limited shortlist of PE and PPE proteins that could be responsible for the essential role of ESX-5 for growth. However, it should be noted that there were more (potential) substrates that did not reach the threshold levels, but showed a trend of partial complementation. In addition, PE and PPE proteins are notoriously difficult to identify using proteomics because of their atypical composition (i.e. many have extremely low number of potential trypsin digestion sites). A recent study showed that also other proteins, not belonging to the PE/PPE protein families, are predicted T7S substrates [40]. One of these predicted T7S substrates, Rv3903c or CpnT, has recently been shown to be an outer membrane porin involved in glycerol uptake [58]. This could be the first example of a T7S substrate that forms an outer membrane porin. However, we were not able to detect CpnT in our cell surface enriched protein extracts or in our cell envelope fractions of *M*. *marinum* [59], suggesting that this protein is not produced in significant amounts in this species. Alternatively, this outer membrane protein is somehow lost during our sample preparations, which could mean that we also miss other outer membrane channels in this analysis. Another recent study on the structure of the ESX-1 substrate EspB gives an interesting insight into possible channel formation of ESX substrates [60], EspB is organized as a ring-shaped heptamer with a central pore and with one side of the ring hydrophobic, suggesting that this protein could form membrane channels. Interestingly the EspB fold is highly similar to PE-PPE, which means that also PE-PPE proteins could form such ring-shaped pores. Our TraDIS analysis indicates that multiple (non-essential) substrates are responsible for the essential phenotype of ESX-5, which makes it difficult to identify them. This is not unexpected, since there are many ESX-5 substrates and they can be divided in major homology groups.

Although the ESX-5 system of *M*. *tuberculosis* can alleviate the essentiality of ESX-5 in *M*. *marinum*, the role of ESX-5 in *M*. *tuberculosis* does not necessarily have to be identical. There have been conflicting results concerning the essentiality of the ESX-5 system in *M*. *tuberculosis*. Di Luca et. al [31] have demonstrated the essentiality of EccB_5_ and EccC_5_ in *M*. *tuberculosis* H37Rv, but in another study they reported that deletion of *eccD*_*5*_ and *espG*_*5*_ (*Rv1794*) [32] was possible in this strain. In contrast, in *M*. *tuberculosis* CDC1551 transposon insertions in *eccC*_*5*_ and *eccD*_*5*_ that block ESX-5 functioning have been described [17]. These results indicate that there are significant differences between different strains of *M*. *tuberculosis*. Based on the results in this study, it can be hypothesized that these differences could also be caused by differences in outer membrane permeability, for instance due to spontaneous mutations in PDIM biogenesis genes. Such mutations have been shown to occur with high frequency in *M*. *tuberculosis*, because they confer a growth advantage in culture [61,62]. In order to investigate whether outer membrane permeability was affected in the CDC1551-derived ESX-5 mutant strains reported in Houben *et al*. [17], we tested the antibiotic sensitivity of these mutant strains and their complemented counterparts. Indeed, we observed a major increase in ampicillin sensitivity as compared to wild-type *M*. *tuberculosis*, in both the *eccC*_*5*_ and *eccD*_*5*_ mutant strains (S5 Fig). Furthermore, the ESX-5 complemented strains still showed this increased susceptibility. However, this increased antibiotic sensitivity was not linked to the absence of PDIMs (S6 Fig). Therefore, probably other factors are responsible for increased outer membrane permeability in these mutants. Based on these results, great care should be taken when interpreting *in vivo* data generated with *esx-5* mutants, because an increase in membrane permeability is known to lead to severe attenuation of virulence [6,61,63], which could lead to wrongfully attributing virulence effects to ESX-5 functioning.

Interestingly, the ESX-5 system is only present in slow-growing mycobacteria [16]. In contrast, the fast-growing species of mycobacteria contain homologues of MspA-like porins that are essential for nutrient uptake [39,64,65]. In previous experiments, it was shown that the expression of MspA is disadvantageous for intracellular survival of fast-growing mycobacteria [63]. From these observations we hypothesize that the ESX-5 system may have evolved to take over the function of this porin in a more selective manner, allowing nutrient import while maintaining the impermeability of the outer membrane required for *in vivo* persistence. Understanding this role of ESX-5 substrates in slow-growing mycobacteria might give insight into the fundamental differences between fast-growing and slow-growing mycobacteria. The essentiality does show that the ESX-5 system is a promising target for novel drug development.

## Materials and Methods

### Bacterial strains and culture conditions

*M*. *marinum* wild-type strain M^VU^ and M^USA^ [30] and its various mutant derivatives were routinely grown in Middlebrook 7H9 liquid medium or Middlebrook 7H10 agar supplemented with 10% Middlebrook ADC or OADC, respectively (BD Biosciences) and 0.05% Tween 80. *E*. *coli* strain DH5α was used for DNA manipulation experiments and propagation of plasmid DNA. When required, antibiotics were added at the following concentration: kanamycin, 25 mg ml-1, hygromycin, 50 mg ml-1 for mycobacteria and 100 mg ml-1 for *E*. *coli* and streptomycin 35 mg ml-1. For growth on defined carbon sources, growth medium was created as described by Capyk et al. [51] and carbon sources were added to a final concentration of 0.2% w/v.

### Construction of plasmids

Anchored primers (MunI for the 5′ primer and HindIII for the 3′ primer; for sequences see S1 Table) were used to amplify both *eccB*_*5*_ and *eccC*_*5*_ from *M*. *marinum* M strain genomic DNA by PCR. Amplicons were cloned as MunI-HindIII fragments in EcoRI-HindIII digested pMV361 [66], resulting in pMV-*eccBC*_*5*_. Similarly, anchored primers (EcoRI for the 5′ primer and HindIII for the 3′ primer) were used to amplify *mycP*_*5*_ from *M*. *marinum* M strain genomic DNA. These amplicons were cloned as EcoRI-HindIII fragments in EcoRI-HindIII digested pMV361, resulting in pMV-*mycP*_*5*_. Subsequently, point mutations were introduced in *eccB*_*5*_ and *eccC*_*5*_ by a nested PCR approach using pMV-*eccBC*_*5*_ as template. In addition, the C-terminal 24 or 1332 amino acids of *eccC*_*5*_ and 363 amino acids of *eccB*_*5*_ were deleted using anchored 3’ primers containing a HindIII restriction site. To create hygromycin versions of the obtained plasmids, the kanamycin resistance cassette was exchanged by a hygromycin resistance cassette by digesting NheI-BcuI. For complementation of the *mas* transposon mutant with a deletion of *eccC*_*5*_, the *mas* gene was amplified from genomic DNA from *M*. *marinum* M using primers Mas-HindIII-F and Mas-R. This product was cloned into the pMV361 vector using HindIII and HpaI, creating pMV-*mas*. In addition, the *mas* gene was isolated from pMV-*mas* by restriction with HindIII and NheI and ligated into pMV-*eccBC*_*5*_ or pMV-*eccBC*_*5*_stop, creating pMV-*eccBC*_*5*_-*mas* and pMV-*eccBC*_*5*_stop-*mas*. For complementation of the *mycP*_*5*_ transposon mutant, pUCintCAT-*mycP*_*5*_ was created by isolating *mycP*_*5*_ from pMV-*mycP*_*5*_ using XbaI and XhoI and ligating it into pUCintCAT-empty [25] using the same enzymes. Finally, a MspA-expression vector was created by amplifying *mspA* from *M*. *smegmatis* chromosomal DNA using anchored primers (NheI for the 5′ primer and BamHI for the 3′ primer) and cloning the obtained amplicon as a NheI-BamHI fragment in pSMT3-LipY [52], digested with the same enzymes. All constructs were checked by sequencing. All used plasmids are listed in S2 Table, while all plasmids used in this study are listed in S4 Table.

### Generating the *eccC*_*5*_, *mycP*_*5*_ and *esx-5* region knock-outs in *M*. *marinum*

An *eccC*_*5*_ and *mycP*_*5*_ knockout was produced in *M*. *marinum* M^VU^ and M^USA^ respectively, by first creating a merodiploid strain. pMV-*eccBC*_*5*_ or pMV-*mycP*_*5*_ was introduced in *M*. *marinum* strains by electroporation, after which endogenous *eccC*_*5*_ or *mycP*_*5*_, respectively, was deleted by allelic exchange using a specialized transducing mycobacteriophage [33]. For deletion of *eccC*_*5*_, fragments bearing the 1164 and 1186 bp of flanking regions of endogenous *eccC*_*5*_ of *M*. *marinum*, which resulted in a deletion of 96% of the gene, were synthesized by PCR (primer set EccC ko Lf and EccC ko Lr for the 5’ region and primer set EccC ko Rf and EccC ko Rr for the 3’ flanking region). For deletion of *mycP*_*5*_, the same procedure was performed with primers MycP5 LF, MycP5 LR, MycP5 RF and MycP5 RR. Amplicons corresponding to 5′ or 3′ flanking regions were digested with AlwNI and cloned into the Van91I digested p0004s plasmid that contains a hygromycin resistance cassette and the *sacB* gene to be able to select for sucrose sensitivity. This allelic exchange substrate was introduced into the PacI site of phasmid phAE159 and electroporated into *M*. *smegmatis* mc^2^155 to obtain high titers of phage pHAE159 according to Bardarov *et al*. [33]. Subsequently, the *M*. *marinum* strain containing pMV-*eccBC*_*5*_ or pMV-*mycP*_*5*_ was incubated with high titers of corresponding phage to create *eccC*_*5*_ and *mycP*_*5*_ delinquents. Colonies in which endogenous *eccC*_*5*_ or *mycP*_*5*_ was deleted were selected on hygromycin plates and verified for sucrose sensitivity. The deletions were confirmed by PCR analysis and sequencing. Using a temperature sensitive phage encoding the γδ-resolvase (TnpR) (a kind gift from Apoorva Bhatt, University of Birmingham, UK), the resistance genes were removed, generating an unmarked deletion mutation. Finally, the pMV-*eccBC*_*5*_ or pMV-*mycP*_*5*_ vectors present in the delinquent strains were exchanged by the plasmids containing the various mutated *eccB*_*5*_, *eccC*_*5*_ and *mycP*_*5*_ genes or no ESX-5 gene (empty vector) and the hygromycin resistance cassette by a switching procedure [34]. In addition, pSMT3-*mspA*, which provides hygromycin resistance, was introduced in the *eccC*_*5*_ and *mycP*_*5*_ delinquent strains, after which the complementing pMV-*eccBC*_*5*_ and pMV-*mycP*_*5*_ vectors were exchanged with pSM128 [67] that integrates at the same site as pMV361 and contains a streptomycin resistance cassette. Colonies that showed proper exchange of the plasmids were subsequently used to introduce kanamycin-resistance conveying plasmids with mutated versions of *eccBC*_5_ and *mycP*_*5*_.

In order to create *Δesx-5*::*mspA* and *Δesx-5*::*esx-5*_*tub*_, another mycobacteriophage was created as described above. For creation of this phage primers esx-5 KO Lf, esx-5 KO Lr, esx-5 KO Rf and esx-5 KO Rr were used to amplify the regions upstream of *eccB*_*5*_ and downstream of *eccA*_*5*_ respectively. These products were cloned, as described above, in a mycobacteriophage vector bearing an apramycin resistance cassette and which lacks the *sacB* counterselection cassette (a kind gift from Apoorva Bhatt, University of Birmingham, UK). We used this newly constructed phage to infect either *M*. *marinum*::*mspA* or the above described *eccC*_*5*_ delinquent strain in which pMV-*eccBC*_*5*_ was first swapped with pMV-*esx-5*_tub_. Deletions were confirmed by PCR and sequencing.

### Generation of the conditional BCG-Pasteur c-*mycP*_*5*_-tet-on and tet-off mutants

For establishing regulated expression of the *mycP*_*5*_ gene, a synthetic gene cassette (hyg-Pmyc1-4XtetO) comprising a hygromycin resistance gene and the Pmyc1 promoter from *M*. *smegmatis* engineered to contain four *tetO* operator sites, which are the DNA binding sites for the cognate repressor protein TetR, was inserted immediately upstream of the *mycP*_*5*_ start codon in *M*. *bovis* BCG-Pasteur (S7 Fig). Targeted gene knock-in was achieved employing the temperature-sensitive mycobacteriophage as described above. For generation of allelic exchange constructs for site-specific insertion in *M*. *bovis* BCG-Pasteur of the hyg-Pmyc1-4X*tetO* cassette, upstream and downstream DNA regions flanking the *mycP*_*5*_ start codon were amplified by PCR employing the primers listed in S1 Table. Subsequently, the upstream and downstream flanks were digested with the indicated restriction enzymes, and ligated with Van91I-digested pcRv1327c-4X*tetO* vector arms. The resulting knock-in plasmid was then linearized cloned and packaged into the temperature-sensitive phage phAE159. The resulting *mycP*_*5*_ knock-in phage was propagated in *M*. *smegmatis* and allelic exchange in *M*. *bovis* BCG-Pasteur was carried out as described above (see also S7 Fig). The obtained BCG-Pasteur knock-in mutant c-*mycP*_*5*_ was verified by Southern analysis of MluI digested genomic DNA using a probe as shown in S7 Fig. For achieving controlled gene expression of the target gene *mycP*_*5*_, either the *E*. *coli* Tn10 *tetR* gene encoding a repressor protein exhibiting high-binding affinity to *tetO* sites in absence of the inducer tetracycline (for establishing a *mycP*_*5*_ tet-on system) or a synthetic gene (*rev-tetR*) derived from Tn10 *tetR* encoding a mutated TetR protein with reversed binding affinity to *tetO* sites upon binding of tetracycline [35] (for establishing a *mycP*_*5*_ tet-off system) was heterologously expressed in the knock-in mutant. For this, the *tetR* gene was amplified by PCR employing the oligonucleotide primer pair TetRFw and TetRRv (S1 Table) using an irrelevant *tetR*-harboring plasmid as a template and cloned using the restriction enzymes EcoRI and HindIII (underlined) into the episomal *E*. *coli*—*mycobacterium* shuttle plasmid pMV261-RBS-E, which is a derivative of plasmid pMV261 [66] harboring a mutated ribosome binding site.

The *rev-tetR* gene was amplified by PCR employing the oligonucleotide primer pair RevTetRFw and RevTetRRv, using the plasmid pTC-28S15-0X (Addgene plasmid 20316, kindly provided by D. Schnappinger) as a template and cloned using the restriction enzymes *EcoR*I and *Hin*dIII (underlined) into the episomal shuttle plasmid pMV261-RBS-F. The resulting plasmids pMV261::*tetR*-RBS-E and pMV261::*rev-tetR*-RBS-F, respectively, providing constitutive gene expression from the HSP60 promoter in mycobacteria were transformed by electroporation into the *M*. *bovis* BCG-Pasteur c-*mycP*_*5*_ knock-in mutant using solid medium containing 50 mg l^-1^ hygromycin and 20 mg l^-1^ kanamycin for selection. This yielded the conditional mutant BCG-Pasteur c-*mycP*_*5*_ pMV261::*tetR*-RBS-E (referred to as BCG-Pasteur c-*mycP5*-tet-on) allowing silencing of the *mycP*_*5*_ gene in absence of the inducer anhydrotetracycline (ATc) or the conditional mutant BCG-Pasteur c-*mycP*_*5*_ pMV261::*rev-tetR*-RBS-F (referred to as BCG-Pasteur c-*mycP*_*5*_-tet-off) allowing silencing of the *mycP*_*5*_ gene in presence of ATc. Due to the operonic organization of *mycP*_*5*_ and the downstream genes *eccE*_*5*_ (*Rv1797*) and *eccA*_*5*_ (*Rv1798*) with transcription likely driven by a single promoter, silencing of *mycP*_*5*_ probably also concomitantly downregulates gene expression of *eccE*_*5*_ *and eccA*_*5*_.

### Viability determination using Alamar Blue

To measure the viability of the BCG-Pasteur c-*mycP*_*5*_-tet-on and-tet-off mutants in liquid culture, metabolic activity was quantified using Alamar Blue dye (Life Technologies) as a correlate of growth in microtiter plates. Cells of the BCG-Pasteur c-*mycP*_*5*_-tet-on and c-*mycP*_*5*_-tet-off mutant were washed and precultured under *mycP*_*5*_ depleting conditions, i.e. in medium containing no ATc for the tet-on strain and in medium containing 10 μg ml^-1^ ATc for the tet-off strain. Subsequently, cultures (total volume 100 μl per well in 96-well plates) containing 50 mgl^-1^ hygromycin, 20 mg l^-1^ kanamycin and increasing concentrations of ATc (0–20 μg ml^-1^) were inoculated 1% (v/v) from the precultures and incubated for 6 days at 37°C. Subsequently, 10% (v/v) Alamar Blue dye solution was added and cells were incubated for a further 16 h at 37°C. Finally, cells were fixed at room temperature for 30 minutes by addition of formalin (5%, v/v, final concentration) and fluorescence was measured using a microplate reader (excitation 560 nm, emission 590 nm).

### Permeability assay

Sensitivity of *M*. *marinum* towards a combination of ampicillin and clavulanic acid was measured by a disc diffusion assay. Bacterial cultures grown to an OD_600_ of 1.0 were diluted 10 times in 0.5% agar, which was kept fluid at 37^°^C. Subsequently, the bacteria-agar suspension was transferred onto 7H10 plates (10 ml per plate) and was let solidify at room temperature. Pills containing 200 μg ampicillin and 25 μg clavulanic acid were placed in the middle of the top-agar and plates were incubated at 30^°^C until a continuous deck of bacterial growth was observed. The surface of growth inhibition of bacteria was measured in cm^2^.

The uptake of ethidium bromide was determined using an adapted protocol as described previously [68]. Bacteria were grown to an OD_600_ of 0.6–1.1. Pellets were collected by centrifugation and resuspended in uptake buffer (50 mM sodium phosphate [pH 7.0], 5 mM magnesium sulfate and 0.05% Tween-80) to an OD_600_ of 0.55 and pre-energized with 25 mM glucose for 5 minutes at room temperature. A final concentration of 20 μM ethidium bromide was added to each sample containing 27 μl of the bacterial suspension and incubated for 60 minutes at room temperature. Samples were washed with PBS containing 0.05% Tween-80 and subsequently acquired on a BD Accuri C6 flow cytometer (BD biosciences) equipped with a 488 nm laser and 585/40 nm filter. 20.000 gated events were collected per sample and data was analyzed using BD CFlow software.

### Transposon mutagenesis, double-filter assay and spotblot analysis

To select for mutations that could rescue the essentiality of *eccC*_*5*_, a transposon library of the *eccC*_*5*_ deletion strain containing the complementation plasmid pMV-*eccBC*_*5*_ was generated using the mycobacterial specific phage phiMycoMarT7 containing the mariner-like transposon Himar1 [69]. Subsequently, transposon mutants, in which *eccC*_*5*_ could be deleted, were selected by exchanging pMV-*eccBC*_*5*_ with empty vector pSM128 as described above [34,67]. The removal of pMV-*eccBC*_*5*_ was confirmed by PCR analysis. To establish the chromosomal location of the transposon insertion, ligation-mediated PCR was used as described by Abdallah *et al*. [30]. Two mutants were identified with transposon insertions at positions 5266 bp and 4072 bp from the transcription start sites of *mas* and *ppsD*, respectively.

Transposon mutagenesis and double filter assays to find PE_PGRS secretion mutants were performed as described in van der Woude *et al*. [29]. In short, strains M^VU^ and M^USA^ were transformed with *pSMT3-mspA* and transposon mutant libraries were created as described above [69]. Libraries were plated out on a nitrocellulose filter (Millipore HATF08250) and grown at 30°C on selective 7H10 plates until colonies were visible. A fresh filter was placed between the original filter and the plate and incubated overnight. The second filter was analyzed on PE_PGRS by antibody labeling, visualized by HRP reduction of 4-Chloronaphtol/3, 3-diaminobenzidine (DAB/CNPO). Colonies on the plates were compared to the stained filters and colonies that did not exhibit staining were rechecked in the double-filter assay. Mutants that exhibited no PE_PGRS secretion in both double-filter assays were tested for surface associated PE_PGRS proteins. For this, 20 mg ml^-1^ wet weight of bacteria was suspended from plate in 0.5% Genapol X-080, vortexed for 1 min and spun down for 5 min at 5000 rpm in a tabletop centrifuge. 2 μl of the detergent supernatant was spotted on a nitrocellulose filter and stained for PE_PGRS proteins as explained above. Only colonies that showed no PE_PGRS proteins on the surface were further analyzed. The transposon integration sites of negative mutants were identified by ligation mediated-PCR as described before [29,30].

### Lipid analysis of phthiocerol dimycocerosates (PDIMs)

*M*. *tuberculosis* and *M*. *marinum* strains were cultured to an OD_600_ of 0.7–1.0 and 50 OD-units biomass was collected. The mycobacterial apolar lipids were extracted according the guidelines published by Minnikin and colleagues [70]. Concisely, the bacterial biomass was treated with a biphasic mixture of methanolic saline and petroleum ether (PE, 60–80°C) and mixed for 1 hour. The biphasic mixture was separated by centrifuging for 10 min at 2000x*g* and the upper PE layer was collected. Subsequently, the lower hydrophilic layer was mixed with PE for a second apolar lipid extraction and the PE layers were combined. The PE layer was N_2_-gas evaporated and subsequently the apolar lipids were re-dissolved in 250 μl dichloromethane. The PDIM analysis was performed with 2D-TLC as described earlier [71]. Briefly, equal amounts of the apolar lipids were spotted on silica TLC plates and PDIM lipids were separated by 2D-TLC solvent system A. The first TLC dimension was performed in solvent PE and ethyl-acetate (98:2) and the second dimension in PE and acetone (98:2). Subsequently, the TLC-plates were air dried and the PDIM lipids were visualized by using 5% ethanolic molybdophosphoric acid (MPA) coloring agent and TLC-plate charring at 150°C for 10 minutes.

### Protein secretion and western blot analysis

Secretion analysis of *M*. *marinum* was performed as described earlier [52]. Briefly, bacterial cultures were grown until mid-logarithmic phase in 7H9 broth supplemented with Middlebrook ADC supplement, 0.05% Tween-80 and appropriate antibiotics. Cells were washed and inoculated in 7H9 without ADC, supplemented with 0.2% dextrose and 0.05% Tween-80 at a starting OD_600_ of 0.35 and incubated overnight, harvested and washed in PBS, while supernatants were filtered through a 0.45 μm filter (Millipore) and TCA precipitated (S). Pellets were resuspended directly in SDS sample buffer (P), or in 100 μl 0.5% Genapol X-080 detergent and incubated for one hour of head-over-head rotation. Cells were spun down and pellets were washed with PBS and resuspended in SDS sample buffer (Genapol pellet (GP)), while 80 μl of the detergent phase was dissolved in 5x concentrated sample buffer (Genapol supernatant (GS)). Western blot of SDS-PAGE gels were stained with polyclonal rabbit sera against EccB_5_ [17], EspG_5_ [17], anti-GroEL2 (kind gift from J. Belisle (Colorado state University and the NIH, Bethesda, MD, USA)) and mouse anti-PE_PGRS (7C4.1F7) [30]. Isolation of cell envelope fractions of *M*. *marinum*, detergent solubilization of these fractions and subsequent Blue Native PAGE and immunoblot analysis were carried out as described previously [17].

### LC-MS/MS

Cell surface proteins of *M*. *marinum* strains M^VU^, M^VU^::*mspA*, M^VU^ *-Δesx-5*::*mspA* and M^VU^-*Δesx-5*::*esx-5*_tub_, were isolated using Genapol X-080 essentially as described above. 100 OD-units of PBS-washed bacteria were resuspended in 10 ml 0.5% Genapol X-080 detergent in PBS and incubated one hour at RT with head-over-head rotation. Bacteria were spun down by low speed centrifugation. Supernatants were collected and extracted proteins were concentrated by TCA precipitation. Cell envelope fractions were isolated from another 100 OD-units of PBS-washed bacteria were isolated as previously described [17]. Samples were analyzed by SDS-PAGE and CBB staining. Total protein lanes were excised in 5 fragments per lane and analyzed by LC-MS/MS [72]. Peptides were separated with a 20 cm x 75 μm ID fused silica C18 column (DrMaisch GMBH, Ammerbuch-Entringen, Germany). Peptides were trapped on a 10 mm x 100 μm ID trap column and separated at 300 nl/min in a 8–32% ACN in 0.5% HAc gradient in 60 min (90 min inject-to-inject). Eluting peptides were ionized at a potential of +2 kVa into a Q Exactive mass spectrometer (Thermo Fisher, Bremen, Germany). Intact masses were measured at resolution 70.000 (at m/z 200) in the orbitrap. MS/MS spectra (top-10 precursors) were acquired at resolution 17.500 (at m/z 200). For protein identification, MS/MS spectra were searched against the Uniprot *M*. *marinum* complete proteome (ATCC BAA-535M) (downloaded march 2013; 5418entries) using MaxQuant 1.3.0.5. [73]. Additionally, for analysis of the *Esx-5*_*tub*_ complementation strain, the FASTA file was supplemented with an *M*. *tuberculosis* ESX-5 locus FASTA file (Rv1782-Rv1798). Enzyme specificity was set to trypsin and up to two missed cleavages were allowed. Cysteine carboxamidomethylation was treated as fixed modification and methionine oxidation and N-terminal acetylation as variable modifications. Peptide precursor ions were searched with a maximum mass deviation of 6 ppm and fragment ions with a maximum mass deviation of 20 ppm. Peptide and protein identifications were filtered at an FDR of 1% using the decoy database strategy. Proteins were (label-free) quantified by spectral counting [74,75], i.e. the sum of all MS/MS spectra for each identified protein. For quantitative analysis across cell-envelope samples, spectral counts were normalized to the sum of the spectral counts per biological sample. Differential analysis of samples was performed using the beta-binominal test, which takes into account within- and between-sample variations, giving fold-change values and associated p-values for all identified proteins [75]. Cell-surface associated samples were defined by comparing normalized spectral counts of the Genapol X-080 samples with those of cell-envelope fractions followed by selecting proteins that were at least four-fold enriched in the Genapol X-080 extracted material. This subset of proteins was then normalized for all samples using ten secreted proteins with a relative stable presence in all samples (pckG, mpt64, fbpA, EsxB_1, MMAR_2949, MMAR_1179, MMAR_0722, MMAR_1553, rpiB & MMAR_2047). This step is required because the overall abundance of proteins in the *esx-5* mutant samples is highly divergent due to the absence of all PE/PPE proteins.

### TraDIS library preparation and sequencing

Construction of TraDIS libraries and sequencing were carried out essentially as described previously [76]. Briefly, about two micrograms of genomic DNA was sheared to an average size of 300 bp. DNA was purified using QiaQuick PCR purification kit (Qiagen) according to the manufacturer’s recommendations, and subsequently Illumina DNA fragment library preparation was performed using NEBNext DNA Library Prep Reagent Set for Illumina (New England BioLabs Inc) following the manufacturer's instructions. Ligated fragments were run in 2% agarose gel, and fragments corresponding to an insert size of 250–350 bp were excised. DNA was extracted from the gel slice using QiaQuick gel extraction kit (Qiagen). To amplify the transposon insertion sites, 22 cycles of PCR were performed using a transposon-specific forward primer and a custom Illumina reverse primer (see Table 1B). Amplified libraries were finally purified with AMPure beads (Beckman Coulter) as per the manufacturer’s instructions. A small aliquot (2 μl) was analyzed on Invitrogen Qubit and Agilent Bioanalyzer DNA1000 chip, following the manufacturer's instructions. The amplified DNA fragment libraries were sequenced on single end Illumina flow cells using an Illumina Genome Analyzer IIx sequencer for 105 cycles of sequencing, using a custom sequencing primer and 2× hybridization buffer. This primer was designed such that the first 10 bp of each read recognizes transposon sequence. Data were processed with the Illumina Pipeline Software v1.82. The TraDIS reads were analysed using the Bio-TraDIS pipeline (https://github.com/sanger-pathogens/Bio-Tradis). The Bio-Tradis pipeline first filters the reads that match the transposon tags. Filtered reads with transposon tags removed are then mapped to M. marinum M-strain reference genome using SMALT (https://www.sanger.ac.uk/resources/software/smalt) short read mapper. Mapped reads were then sorted using samtools. Insertion sites plots were analyzed by TraDIS essentiality R script to obtain a list of essential genes. All primers used for the TraDIS analysis are listed in S3 Table.

### Fatty acid uptake experiments

Indicated *M*. *marinum* strains were grown to mid-logarithmic phase, washed and 0.5 OD units were inoculated in 2 ml screw caps containing 1.5 ml 7H9 medium containing 0.05% Tween-80. To measure fatty acid uptake, 4μg/ml fluorescently labeled fatty acid Bodipy 558/568 C_12_ (Life Technologies) was added to three independent cultures. Two cultures were incubated without fluorescently labeled fatty acid. All cultures were grown for 72 hours under hypoxic conditions, after which Bodipy-C_12_ was added to one of the control cultures and incubated one more hour as a negative control. 1 ml of all cultures was washed with PBS, and acquired after gating for similar particle size on a BD Accuri C6 flow cytometer (BD biosciences) as described above. Mean fluorescent intensity was calculated for all samples and adjusted for negative controls. One-way ANOVA was performed to analyze statistical differences between the groups. 0.5 ml of the cultures were additionally washed with PBS and fixed in 4% paraformaldehyde. Bacteria were dried on glass slides and analyzed by confocal microscopy (Leica TCS SP8). Imaging was performed using Leica confocal software with identical settings for each strain.

## Supporting Information

S1 Fig 2D-TLC analysis of apolar lipids of *M*. *marinum mas* and *eccC*_*5*_ mutants. Apolar lipids were extracted from the various *M*. *marinum* strains and analyzed for the presence of PDIMs by 2D-TLC. The first dimension consisted of PE:ethyl ether 98:2, the second dimension of PE:acetone 98:2. Wild-type strains M^VU^ (A) and M^USA^ (C) showed the presence of PDIM indicated by black arrows. No PDIMs could be detected in an *mas*::*tn* mutant picked up in an independent transposon screen (B), in a targeted knock-out of *eccC*_*5*_ combined with a transposon insertion in *mas*, picked up in our transposon screen (*mas*::*tn*-*ΔeccC*_*5*_; D) and the complementant of this latter strain (*mas*::*tn*-*ΔeccC*_*5*_-C; E).
(TIF)

S2 Fig Expression of MspA has no effects on PDIM expression. *M*. *marinum*::*mspA-ΔeccC*_*5*_::pMV::*eccBC*_*5*_ (A), *M*. *marinum*::*mspA-ΔeccC*_*5*_::pMV361 (B) and an independent PDIM negative *ppsD*::*tn* mutant (C) were analyzed for the presence of PDIMs by 2D-TLC. 1D = PE:ethyl ether 98:2, 2D = PE:acetone 98:2. PDIMs are indicated by black arrows.
(TIF)

S3 Fig Phenotype of *M*. *marinum espG*_*5*_ and *eccB*_*5*_ transposon mutants. *M*. *marinum*::*mspA* and transposon mutants LA1 (*espG*_*5*_::*tn*) and LA2 (*eccB*_*5*_::*tn*) were analyzed for their expression and secretion of PE-PGRS proteins (A) and expression of EccB_5_ and EspG_5_ (B) by immunoblotting. Bacterial pellets were incubated with Genapol X-080, after which non-extracted material (GP) was separated by the solubilized material (GS) by centrifugation. Non-treated bacterial pellets (P) were additionally analyzed for the expression of EccB_5_ and EspG_5_ (B).
(TIF)

S4 Fig Growth of *M*. *marinum* on different carbon sources. A) Growth of indicated *M*. *marinum* strains on 0.2% glucose, acetate or glycerol as a sole carbon source was assessed by measuring optical density at different time points. B) Growth of indicated strains at Tween-40. C) *M*. *marinum* Δ*mycP5*::*mspA* cannot grow on Tween-80 as a sole carbon source, but is able to grow normally when 0.2% glucose was added at day 8 (indicated by the arrow). D) *M*. *marinum*::pSMT3-*crimson* is not able to rescue growth of the *mycP*_*5*_ mutant. A mixed culture of the two indicated strains on Tween-80 as a sole carbon source was performed. On the indicated time points bacteria were plated out on 7H10 plates with hygromycin and colony forming units (CFU) were quantified by counting fluorescent (WT) and non-fluorescent (Δ*mycP*_*5*_) colonies. E) *M*. *marinum* Δ*lipY* can grow normally on Tween-80 as a sole carbon source. In B-D, error bars depict the standard deviation over three independent cultures.
(TIF)

S5 Fig Ampicillin susceptibility of *M*. *tuberculosis* CDC1551 ESX-5 mutants. *M*. *tuberculosis* H37Rv and CDC1551 wild-type strains, CDC1551 derived transposon mutants in *eccC*_*5*_ (*eccC*_*5*_::*tn*) or *eccD*_*5*_ (*eccD*_*5*_::*tn*); and their respective complemented strains (*eccC*_*5*_::*tn*-C and *eccD*_*5*_::*tn*-C), were grown on 7H10 plates containing a combination of 30μg/ml ampicillin and 30μg/ml clavulanic acid (A), or without antibiotics (B). 5 μl of a 1.0 OD_600_ culture was spotted in serial dilutions (top row, undiluted; second row, 10x diluted; third row 100x diluted). In the absence of antibiotics, the H37Rv strain grew faster than the CDC1551 strains, while the *eccC*_*5*_::*tn* strain showed a growth defect, which was alleviated upon complementation. Both ESX-5 transposon mutants and their respective complementants did not grow in the presence of 30μg/ml ampicillin and 30μg/ml clavulanic acid, indicating increased membrane permeability in these strains compared to the wild-type strain.
(TIF)

S6 Fig PDIMs are present in *M*. *tuberculosis* CDC1551 *esx-5* mutants. Apolar lipids were extracted from *M*. *tuberculosis* CDC1551 (A), and transposon mutants *eccC*_*5*_::*tn* (*rv1783*, B) and *eccD*_*5*_::*tn* (*rv1795*, C) as described before and were analyzed for the presence of PDIMs by 2D-TLC. 1D = PE:ethyl ether 98:2, 2D = PE:acetone 98:2. PDIMs are indicated by black arrows. PDIMs are visible in all strains, although the *eccC*_*5*_ mutant appears to make higher amounts of PDIMs.
(TIF)

S7 Fig Construction of an inducible MycP_5_ expression system in *M*. *bovis* BCG. Construction of the *M*. *bovis* BCG-Pasteur c-*mycP*_*5*_-Tet-on mutant. A) Organization of the *mycP*_*5*_ locus in *M*. *bovis* BCG-Pasteur wild-type (WT) and the *mycP*_*5*_ knock-in mutant. The sizes of relevant fragments as well as the location of the probe used for Southern blot analysis are indicated. Hyg, hygromycin resistance gene; *Pmyc1-4×tetO*, modified *Pmyc1* promoter harboring 4 *tetO* sites. The same method was used to construct c-*mycP*_*5*_-Tet-off, for which 4 synthetic tet-repressor sites (*tetR*) were introduced instead of the *tetO* sites. B) Southern blot analysis of MluI-digested genomic DNA using a probe hybridizing to the position indicated in (A), showing knock-in of the promoter cassette in c-*mycP*_*5*_-Tet-on.
(TIF)

S1 Table ESX-5-dependent Cell envelope proteins of *M*.*marinum*. Cell envelope proteins of *M*. *marinum*::*mspA*, *Δesx-5*::*mspA* and *esx-5*_*tub*_ strains were analyzed by LC-MS/MS. Proteins were classified as ESX-5-dependent when the normalized spectral counts between *M*. *marinum*::*mspA* and *Δesx-5*::*mspA* were reduced at least 10-fold. Grey highlighted rows indicate the conserved ESX-5 components.
(DOCX)

S2 Table List of primers used for cloning in this study. (DOCX)

S3 Table Primers used for TraDIS experiments. (DOCX)

S4 Table List of plasmids used in this study. (DOCX)

S1 File LC-MS/MS results of cell surface and cell envelope fractions. (XLS)

S2 File TraDIS results of genes that have more transposon insertions upon expression of *mspA*. (XLS)

## References

[1] World Health Organization (2013) Global tuberculosis report 2013.

[2] Goodfellow & Jones (2012) Bergey’s Manual of Systematic Bacteriology: Volume 5: The Actinobacteria, Volume 5, Parts 1–2. Springer.

[3] Alibaud L, Pawelczyk J, Gannoun-Zaki L, Singh VK, Rombouts Y, et al (2013) Increased phagocytosis of *Mycobacterium marinum* mutants defective in lipooligosaccharide production: a structure-activity relationship study. J Biol Chem 289: 215–228. 10.1074/jbc.M113.525550 24235141 PMC3879545

[4] Brennan PJ, Nikaido H (1995) The envelope of mycobacteria. Annu Rev Biochem 64: 29–63. 7574484 10.1146/annurev.bi.64.070195.000333

[5] Camacho LR, Ensergueix D, Perez E, Gicquel B, Guilhot C (1999) Identification of a virulence gene cluster of *Mycobacterium tuberculosis* by signature-tagged transposon mutagenesis. Mol Microbiol 34: 257–267. 10564470 10.1046/j.1365-2958.1999.01593.x

[6] Yu J, Tran V, Li M, Huang X, Niu C, et al (2012) Both phthiocerol dimycocerosates and phenolic glycolipids are required for virulence of *Mycobacterium marinum* . Infect Immun 80: 1381–1389. 10.1128/IAI.06370-11 22290144 PMC3318414

[7] Hunter RL, Armitige L, Jagannath C, Actor JK (2009) TB research at UT-Houston—a review of cord factor: new approaches to drugs, vaccines and the pathogenesis of tuberculosis. Tuberculosis (Edinb) 89 Suppl 1: S18–25. 20006299 10.1016/S1472-9792(09)70007-1 PMC3682682

[8] Hoffmann C, Leis A, Niederweis M, Plitzko JM, Engelhardt H (2008) Disclosure of the mycobacterial outer membrane: cryo-electron tomography and vitreous sections reveal the lipid bilayer structure. Proc Natl Acad Sci U S A 105: 3963–3967. 10.1073/pnas.0709530105 18316738 PMC2268800

[9] Zuber B, Chami M, Houssin C, Dubochet J, Griffiths G, et al (2008) Direct visualization of the outer membrane of mycobacteria and corynebacteria in their native state. J Bacteriol 190: 5672–5680. 10.1128/JB.01919-07 18567661 PMC2519390

[10] Purdy GE, Niederweis M, Russell DG (2009) Decreased outer membrane permeability protects mycobacteria from killing by ubiquitin-derived peptides. Mol Microbiol 73: 844–857. 10.1111/j.1365-2958.2009.06801.x 19682257 PMC2747030

[11] Gao L-Y, Laval F, Lawson EH, Groger RK, Woodruff A, et al (2003) Requirement for kasB in Mycobacterium mycolic acid biosynthesis, cell wall impermeability and intracellular survival: implications for therapy. Mol Microbiol 49: 1547–1563. 12950920 10.1046/j.1365-2958.2003.03667.x

[12] Stoop EJM, Bitter W, van der Sar AM (2012) Tubercle bacilli rely on a type VII army for pathogenicity. Trends Microbiol 20: 477–484. 10.1016/j.tim.2012.07.001 22858229

[13] Simeone R, Bottai D, Brosch R (2009) ESX/type VII secretion systems and their role in host-pathogen interaction. Curr Opin Microbiol 12: 4–10. 10.1016/j.mib.2008.11.003 19155186

[14] Houben ENG, Korotkov K V, Bitter W (2013) Take five—Type VII secretion systems of Mycobacteria. Biochim Biophys Acta 1843: 1707–1716. 10.1016/j.bbamcr.2013.11.003 24263244

[15] Bitter W, Houben ENG, Bottai D, Brodin P, Brown EJ, et al (2009) Systematic genetic nomenclature for type VII secretion systems. PLoS Pathog 5: e1000507 10.1371/journal.ppat.1000507 19876390 PMC2763215

[16] Gey van Pittius NC, Sampson SL, Lee H, Kim Y, van Helden PD, et al (2006) Evolution and expansion of the *Mycobacterium tuberculosis* PE and PPE multigene families and their association with the duplication of the ESAT-6 (esx) gene cluster regions. BMC Evol Biol 6: 95 17105670 10.1186/1471-2148-6-95 PMC1660551

[17] Houben ENG, Bestebroer J, Ummels R, Wilson L, Piersma SR, et al (2012) Composition of the type VII secretion system membrane complex. Mol Microbiol 86: 472–484. 10.1111/j.1365-2958.2012.08206.x 22925462

[18] Champion PAD, Stanley SA, Champion MM, Brown EJ, Cox JS (2006) C-terminal signal sequence promotes virulence factor secretion in *Mycobacterium tuberculosis* . Science 313: 1632–1636. 16973880 10.1126/science.1131167

[19] Pym AS, Brodin P, Majlessi L, Brosch R, Demangel C, et al (2003) Recombinant BCG exporting ESAT-6 confers enhanced protection against tuberculosis. Nat Med 9: 533–539. 12692540 10.1038/nm859

[20] Ohol YM, Goetz DH, Chan K, Shiloh MU, Craik CS, et al (2010) *Mycobacterium tuberculosis* MycP1 protease plays a dual role in regulation of ESX-1 secretion and virulence. Cell Host Microbe 7: 210–220. 10.1016/j.chom.2010.02.006 20227664 PMC3121311

[21] Houben D, Demangel C, van Ingen J, Perez J, Baldeón L, et al (2012) ESX-1-mediated translocation to the cytosol controls virulence of mycobacteria. Cell Microbiol 14: 1287–1298. 10.1111/j.1462-5822.2012.01799.x 22524898

[22] Simeone R, Bobard A, Lippmann J, Bitter W, Majlessi L, et al (2012) Phagosomal rupture by *Mycobacterium tuberculosis* results in toxicity and host cell death. PLoS Pathog 8: e1002507 10.1371/journal.ppat.1002507 22319448 PMC3271072

[23] Siegrist MS, Unnikrishnan M, McConnell MJ, Borowsky M, Cheng T-Y, et al (2009) Mycobacterial Esx-3 is required for mycobactin-mediated iron acquisition. Proc Natl Acad Sci U S A 106: 18792–18797. 10.1073/pnas.0900589106 19846780 PMC2774023

[24] Serafini A, Boldrin F, Palù G, Manganelli R (2009) Characterization of a *Mycobacterium tuberculosis* ESX-3 conditional mutant: essentiality and rescue by iron and zinc. J Bacteriol 191: 6340–6344. 10.1128/JB.00756-09 19684129 PMC2753049

[25] Abdallah AM, Verboom T, Weerdenburg EM, Gey van Pittius NC, Mahasha PW, et al (2009) PPE and PE_PGRS proteins of *Mycobacterium marinum* are transported via the type VII secretion system ESX-5. Mol Microbiol 73: 329–340. 10.1111/j.1365-2958.2009.06783.x 19602152

[26] Iantomasi R, Sali M, Cascioferro A, Palucci I, Zumbo A, et al (2012) PE_PGRS30 is required for the full virulence of *Mycobacterium tuberculosis* . Cell Microbiol 14: 356–367. 10.1111/j.1462-5822.2011.01721.x 22050772

[27] Chaturvedi R, Bansal K, Narayana Y, Kapoor N, Sukumar N, et al (2010) The multifunctional PE_PGRS11 protein from *Mycobacterium tuberculosis* plays a role in regulating resistance to oxidative stress. J Biol Chem 285: 30389–30403. 10.1074/jbc.M110.135251 20558725 PMC2945531

[28] Abdallah AM, Savage NDL, van Zon M, Wilson L, Vandenbroucke-Grauls CMJE, et al (2008) The ESX-5 secretion system of *Mycobacterium marinum* modulates the macrophage response. J Immunol 181: 7166–7175. 18981138 10.4049/jimmunol.181.10.7166

[29] Van der Woude AD, Sarkar D, Bhatt A, Sparrius M, Raadsen S a, et al (2012) Unexpected link between lipooligosaccharide biosynthesis and surface protein release in *Mycobacterium marinum* . J Biol Chem 287: 20417–20429. 10.1074/jbc.M111.336461 22505711 PMC3370222

[30] Abdallah AM, Verboom T, Hannes F, Safi M, Strong M, et al (2006) A specific secretion system mediates PPE41 transport in pathogenic mycobacteria. Mol Microbiol 62: 667–679. 17076665 10.1111/j.1365-2958.2006.05409.x

[31] Di Luca M, Bottai D, Batoni G, Orgeur M, Aulicino A, et al (2012) The ESX-5 associated eccB-EccC locus is essential for *Mycobacterium tuberculosis* viability. PLoS One 7: e52059 10.1371/journal.pone.0052059 23284869 PMC3524121

[32] Bottai D, Di Luca M, Majlessi L, Frigui W, Simeone R, et al (2012) Disruption of the ESX-5 system of *Mycobacterium tuberculosis* causes loss of PPE protein secretion, reduction of cell wall integrity and strong attenuation. Mol Microbiol 83: 1195–1209. 10.1111/j.1365-2958.2012.08001.x 22340629

[33] Bardarov S, Kriakov J, Carriere C, Yu S, Vaamonde C, et al (1997) Conditionally replicating mycobacteriophages: a system for transposon delivery to *Mycobacterium tuberculosis* . Proc Natl Acad Sci U S A 94: 10961–10966. 9380742 10.1073/pnas.94.20.10961 PMC23545

[34] Pashley CA, Parish T (2003) Efficient switching of mycobacteriophage L5-based integrating plasmids in *Mycobacterium tuberculosis* . FEMS Microbiol Lett 229: 211–215. 14680701 10.1016/S0378-1097(03)00823-1

[35] Klotzsche M, Ehrt S, Schnappinger D (2009) Improved tetracycline repressors for gene silencing in mycobacteria. Nucleic Acids Res 37: 1778–1788. 10.1093/nar/gkp015 19174563 PMC2665214

[36] Jones CM, Niederweis M (2010) Role of porins in iron uptake by *Mycobacterium smegmatis* . J Bacteriol 192: 6411–6417. 10.1128/JB.00986-10 20952578 PMC3008526

[37] Stephan J, Bender J, Wolschendorf F, Hoffmann C, Roth E, et al (2005) The growth rate of *Mycobacterium smegmatis* depends on sufficient porin-mediated influx of nutrients. Mol Microbiol 58: 714–730. 16238622 10.1111/j.1365-2958.2005.04878.x

[38] Stahl C, Kubetzko S, Kaps I, Seeber S, Engelhardt H, et al (2001) MspA provides the main hydrophilic pathway through the cell wall of *Mycobacterium smegmatis* . Mol Microbiol 40: 451–464. 11309127 10.1046/j.1365-2958.2001.02394.x

[39] Stephan J, Mailaender C, Etienne G, Daffé M, Niederweis M (2004) Multidrug resistance of a porin deletion mutant of *Mycobacterium smegmatis* . Antimicrob Agents Chemother 48: 4163–4170. 15504836 10.1128/AAC.48.11.4163-4170.2004 PMC525411

[40] Moncalián G, Cabezón E, Alkorta I, Valle M, Moro F, et al (1999) Characterization of ATP and DNA binding activities of TrwB, the coupling protein essential in plasmid R388 conjugation. J Biol Chem 274: 36117–36124. 10593894 10.1074/jbc.274.51.36117

[41] Cascales E, Christie PJ (2004) Agrobacterium VirB10, an ATP energy sensor required for type IV secretion. Proc Natl Acad Sci U S A 101: 17228–17233. 15569944 10.1073/pnas.0405843101 PMC535377

[42] Daleke MH, van der Woude AD, Parret AH a, Ummels R, de Groot a M, et al (2012) Specific chaperones for the type VII protein secretion pathway. J Biol Chem 287: 31939–31947. 10.1074/jbc.M112.397596 22843727 PMC3442526

[43] Sani M, Houben ENG, Geurtsen J, Pierson J, de Punder K, et al (2010) Direct visualization by cryo-EM of the mycobacterial capsular layer: a labile structure containing ESX-1-secreted proteins. PLoS Pathog 6: e1000794 10.1371/journal.ppat.1000794 20221442 PMC2832766

[44] Cascioferro A, Delogu G, Colone M, Sali M, Stringaro A, et al (2007) PE is a functional domain responsible for protein translocation and localization on mycobacterial cell wall. Mol Microbiol 66: 1536–1547. 18028308 10.1111/j.1365-2958.2007.06023.x

[45] Kapopoulou A, Lew JM, Cole ST (2011) The MycoBrowser portal: a comprehensive and manually annotated resource for mycobacterial genomes. Tuberculosis (Edinb) 91: 8–13. 10.1016/j.tube.2010.09.006 20980200

[46] Weerdenburg EM, Abdallah AM, Rangkuti F, Abd El Ghany M, Otto TD, et al (2015) Genome-wide transposon mutagenesis indicates that *Mycobacterium marinum* customizes its virulence mechanisms for survival and replication in different hosts. Infect Immun: IAI.03050–14. 25690095 10.1128/IAI.03050-14 PMC4399070

[47] Daleke MH, Ummels R, Bawono P, Heringa J, Vandenbroucke-Grauls CMJE, et al (2012) General secretion signal for the mycobacterial type VII secretion pathway. Proc Natl Acad Sci U S A 109: 11342–11347. 10.1073/pnas.1119453109 22733768 PMC3396530

[48] Griffin JE, Gawronski JD, Dejesus MA, Ioerger TR, Akerley BJ, et al (2011) High-resolution phenotypic profiling defines genes essential for mycobacterial growth and cholesterol catabolism. PLoS Pathog 7: e1002251 10.1371/journal.ppat.1002251 21980284 PMC3182942

[49] Pritchard JR, Chao MC, Abel S, Davis BM, Baranowski C, et al (2014) ARTIST: High-Resolution Genome-Wide Assessment of Fitness Using Transposon-Insertion Sequencing. PLoS Genet 10: e1004782 10.1371/journal.pgen.1004782 25375795 PMC4222735

[50] Eoh H, Rhee KY (2014) Methylcitrate cycle defines the bactericidal essentiality of isocitrate lyase for survival of *Mycobacterium tuberculosis* on fatty acids. Proc Natl Acad Sci U S A 111: 4976–4981. 10.1073/pnas.1400390111 24639517 PMC3977286

[51] Capyk JK, Kalscheuer R, Stewart GR, Liu J, Kwon H, et al (2009) Mycobacterial cytochrome p450 125 (cyp125) catalyzes the terminal hydroxylation of c27 steroids. J Biol Chem 284: 35534–35542. 10.1074/jbc.M109.072132 19846551 PMC2790983

[52] Daleke MH, Cascioferro A, de Punder K, Ummels R, Abdallah AM, et al (2011) Conserved Pro-Glu (PE) and Pro-Pro-Glu (PPE) protein domains target LipY lipases of pathogenic mycobacteria to the cell surface via the ESX-5 pathway. J Biol Chem 286: 19024–19034. 10.1074/jbc.M110.204966 21471225 PMC3099717

[53] Daniel J, Maamar H, Deb C, Sirakova TD, Kolattukudy PE (2011) *Mycobacterium tuberculosis* uses host triacylglycerol to accumulate lipid droplets and acquires a dormancy-like phenotype in lipid-loaded macrophages. PLoS Pathog 7: e1002093 10.1371/journal.ppat.1002093 21731490 PMC3121879

[54] Sirakova TD, Dubey VS, Deb C, Daniel J, Korotkova TA, et al (2006) Identification of a diacylglycerol acyltransferase gene involved in accumulation of triacylglycerol in *Mycobacterium tuberculosis* under stress. Microbiology 152: 2717–2725. 16946266 10.1099/mic.0.28993-0 PMC1575465

[55] Weerdenburg EM, Abdallah AM, Mitra S, de Punder K, van der Wel NN, et al (2012) ESX-5-deficient *Mycobacterium marinum* is hypervirulent in adult zebrafish. Cell Microbiol 14: 728–739. 10.1111/j.1462-5822.2012.01755.x 22256857

[56] Camacho LR, Constant P, Raynaud C, Laneelle MA, Triccas JA, et al (2001) Analysis of the phthiocerol dimycocerosate locus of *Mycobacterium tuberculosis*. Evidence that this lipid is involved in the cell wall permeability barrier. J Biol Chem 276: 19845–19854. 11279114 10.1074/jbc.M100662200

[57] Sampson SL (2011) Mycobacterial PE/PPE proteins at the host-pathogen interface. Clin Dev Immunol 2011: 497203 10.1155/2011/497203 21318182 PMC3034920

[58] Danilchanka O, Sun J, Pavlenok M, Maueröder C, Speer A, et al (2014) An outer membrane channel protein of *Mycobacterium tuberculosis* with exotoxin activity. Proc Natl Acad Sci U S A 111: 6750–6755. 10.1073/pnas.1400136111 24753609 PMC4020113

[59] Van der Woude AD, Mahendran KR, Ummels R, Piersma SR, Pham TV, et al (2013) Differential detergent extraction of *Mycobacterium marinum* cell envelope proteins identifies an extensively modified threonine-rich outer membrane protein with channel activity. J Bacteriol 195: 2050–2059. 10.1128/JB.02236-12 23457249 PMC3624596

[60] Solomonson M, Setiaputra D, Makepeace KAT, Lameignere E, Petrotchenko E V, et al (2015) Structure of EspB from the ESX-1 Type VII secretion system and insights into its export mechanism. Structure, 23: 571–583. 10.1016/j.str.2015.01.002 25684576

[61] Kirksey MA, Tischler AD, Siméone R, Hisert KB, Uplekar S, et al (2011) Spontaneous phthiocerol dimycocerosate-deficient variants of *Mycobacterium tuberculosis* are susceptible to gamma interferon-mediated immunity. Infect Immun 79: 2829–2838. 10.1128/IAI.00097-11 21576344 PMC3191967

[62] Domenech P, Reed MB (2009) Rapid and spontaneous loss of phthiocerol dimycocerosate (PDIM) from *Mycobacterium tuberculosis* grown in vitro: implications for virulence studies. Microbiology 155: 3532–3543. 10.1099/mic.0.029199-0 19661177 PMC5154741

[63] Sharbati-Tehrani S, Stephan J, Holland G, Appel B, Niederweis M, et al (2005) Porins limit the intracellular persistence of *Mycobacterium smegmatis* . Microbiology 151: 2403–2410. 16000730 10.1099/mic.0.27969-0

[64] Wolschendorf F, Mahfoud M, Niederweis M (2007) Porins are required for uptake of phosphates by *Mycobacterium smegmatis* . J Bacteriol 189: 2435–2442. 17209034 10.1128/JB.01600-06 PMC1899398

[65] Niederweis M, Ehrt S, Heinz C, Klöcker U, Karosi S, et al (1999) Cloning of the mspA gene encoding a porin from *Mycobacterium smegmatis* . Mol Microbiol 33: 933–945. 10476028 10.1046/j.1365-2958.1999.01472.x

[66] Stover CK, de la Cruz VF, Fuerst TR, Burlein JE, Benson LA, et al (1991) New use of BCG for recombinant vaccines. Nature 351: 456–460. 1904554 10.1038/351456a0

[67] Timm J, Lim EM, Gicquel B (1994) *Escherichia coli*-mycobacteria shuttle vectors for operon and gene fusions to lacZ: the pJEM series. J Bacteriol 176: 6749–6753. 7961429 10.1128/jb.176.21.6749-6753.1994 PMC197033

[68] Danilchanka O, Mailaender C, Niederweis M (2008) Identification of a novel multidrug efflux pump of *Mycobacterium tuberculosis* . Antimicrob Agents Chemother 52: 2503–2511. 10.1128/AAC.00298-08 18458127 PMC2443884

[69] Sassetti CM, Boyd DH, Rubin EJ (2003) Genes required for mycobacterial growth defined by high density mutagenesis. Mol Microbiol 48: 77–84. 12657046 10.1046/j.1365-2958.2003.03425.x

[70] Minnikin D., Dobson G, Parlett JH (1985) Extraction and Chromatographic Analysis of Characteristic Mycobacterial Lipids In: Habermehl KO, editor. Rapid Methods and Automation in Microbiology and Immunology. Berlin/Heidelberg: Springer Verlag pp. 274–282

[71] Besra GS (1998) Preparation of cell-wall fractions from mycobacteria In: Parish T, Stoker NG, editors. Mycobacteria protocols. New York: Humana Press pp. 91–107 10.1385/0-89603-471-2:91 9921472

[72] Piersma SR, Warmoes MO, de Wit M, de Reus I, Knol JC, et al (2013) Whole gel processing procedure for GeLC-MS/MS based proteomics. Proteome Sci 11: 17 10.1186/1477-5956-11-17 23617947 PMC3656797

[73] Cox J, Mann M (2008) MaxQuant enables high peptide identification rates, individualized p.p.b.-range mass accuracies and proteome-wide protein quantification. Nat Biotechnol 26: 1367–1372. 10.1038/nbt.1511 19029910

[74] Liu H, Sadygov RG, Yates JR (2004) A model for random sampling and estimation of relative protein abundance in shotgun proteomics. Anal Chem 76: 4193–4201. 15253663 10.1021/ac0498563

[75] Pham T V, Piersma SR, Warmoes M, Jimenez CR (2010) On the beta-binomial model for analysis of spectral count data in label-free tandem mass spectrometry-based proteomics. Bioinformatics 26: 363–369. 10.1093/bioinformatics/btp677 20007255

[76] Langridge GC, Phan M-D, Turner DJ, Perkins TT, Parts L, et al (2009) Simultaneous assay of every *Salmonella typhi* gene using one million transposon mutants. Genome Res 19: 2308–2316. 10.1101/gr.097097.109 19826075 PMC2792183

