# Correction: Essential Role of the ESX-5 Secretion System in Outer Membrane Permeability of Pathogenic Mycobacteria

**DOI:** 10.1371/journal.pgen.1005270

**Published:** 2015-05-29

**Authors:** The PLOS Genetics Staff

The forth author’s name is spelled incorrectly. The correct name is: Robert van de Weerd. The correct citation is: Ates LS, Ummels R, Commandeur S, van de Weerd R, Sparrius M, Weerdenburg E, et al. (2015) Essential Role of the ESX-5 Secretion System in Outer Membrane Permeability of Pathogenic Mycobacteria. PLoS Genet 11(5): e1005190. doi:10.1371/journal.pgen.1005190

## References

[1] Ates LS, Ummels R, Commandeur S, van der Weerd R, Sparrius M, Weerdenburg E, et al (2015) Essential Role of the ESX-5 Secretion System in Outer Membrane Permeability of Pathogenic Mycobacteria. PLoS Genet 11(5): e1005190 doi:10.1371/journal.pgen.1005190 25938982 10.1371/journal.pgen.1005190 PMC4418733

