# Supplementary figures and images for: Essential Role of the ESX-5 Secretion System in Outer Membrane Permeability of Pathogenic Mycobacteria
Source: PLoS Genet. 2015 May 4;11(5):e1005190. doi: 10.1371/journal.pgen.1005190 (PMC4418733; doi:10.1371/journal.pgen.1005190)

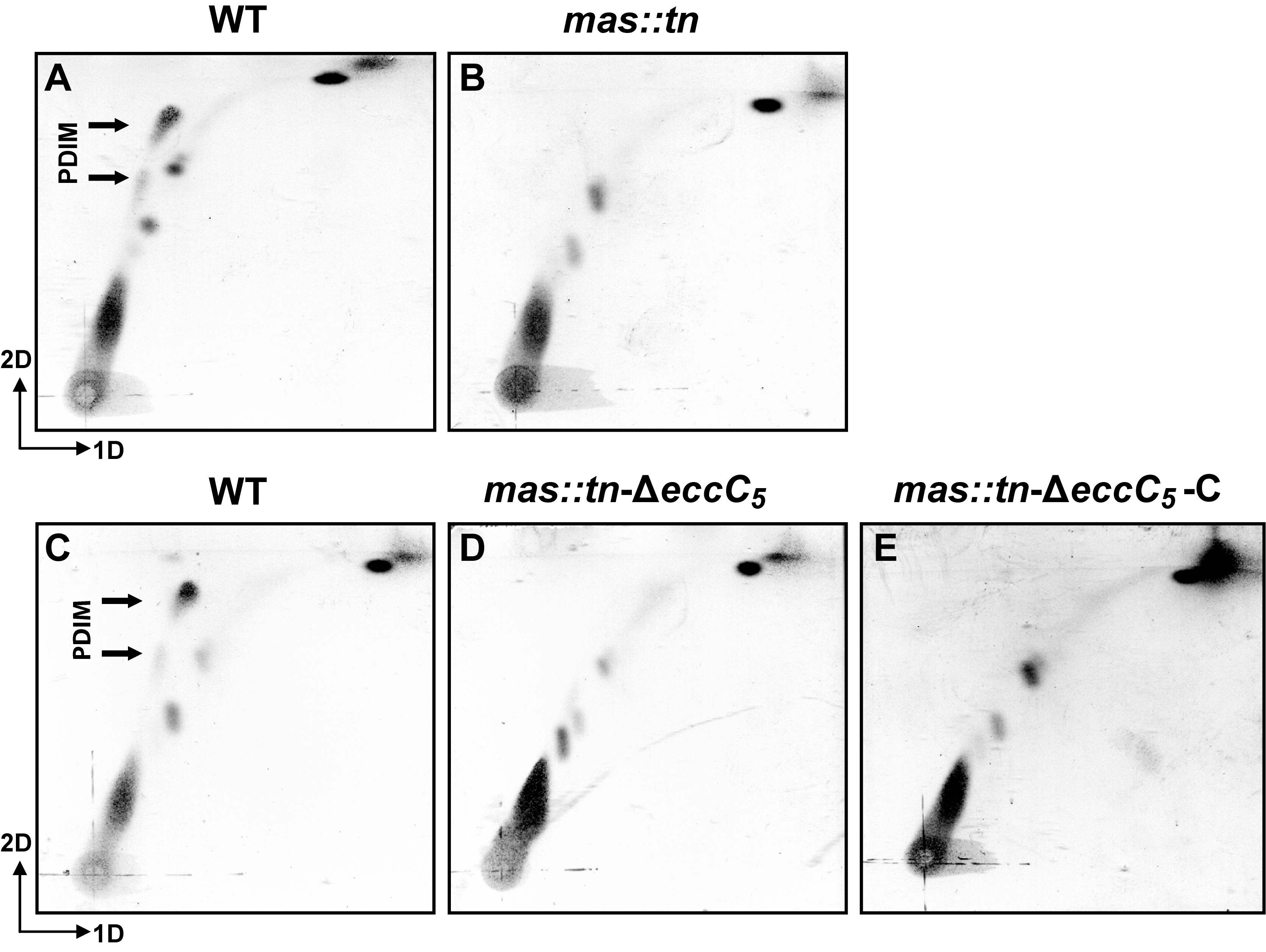

Supplement: S1 Fig — Apolar lipids were extracted from the various M. marinum strains and analyzed for the presence of PDIMs by 2D-TLC. The first dimension consisted of PE:ethyl ether 98:2, the second dimension of PE:acetone 98:2. Wild-type strains MVU (A) and MUSA (C) showed the presence of PDIM indicated by black arrows. No PDIMs could be detected in an mas::tn mutant picked up in an independent transposon screen (B), in a targeted knock-out of eccC 5 combined with a transposon insertion in mas, picked up in our transposon screen (mas::tn-ΔeccC 5; D) and the complementant of this latter strain (mas::tn-ΔeccC 5-C; E). (TIF) [file pgen.1005190.s001.tif]

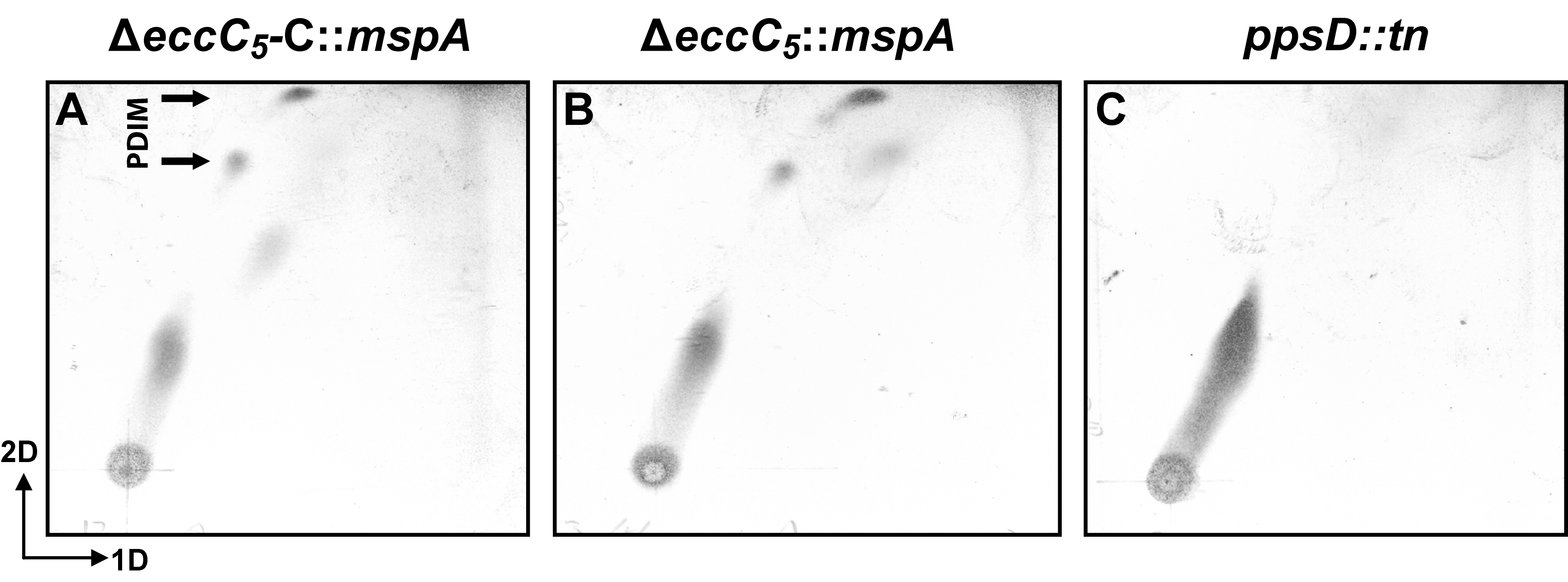

Supplement: S2 Fig — M. marinum::mspA-ΔeccC 5::pMV::eccBC 5 (A), M. marinum::mspA-ΔeccC 5::pMV361 (B) and an independent PDIM negative ppsD::tn mutant (C) were analyzed for the presence of PDIMs by 2D-TLC. 1D = PE:ethyl ether 98:2, 2D = PE:acetone 98:2. PDIMs are indicated by black arrows. (TIF) [file pgen.1005190.s002.tif]

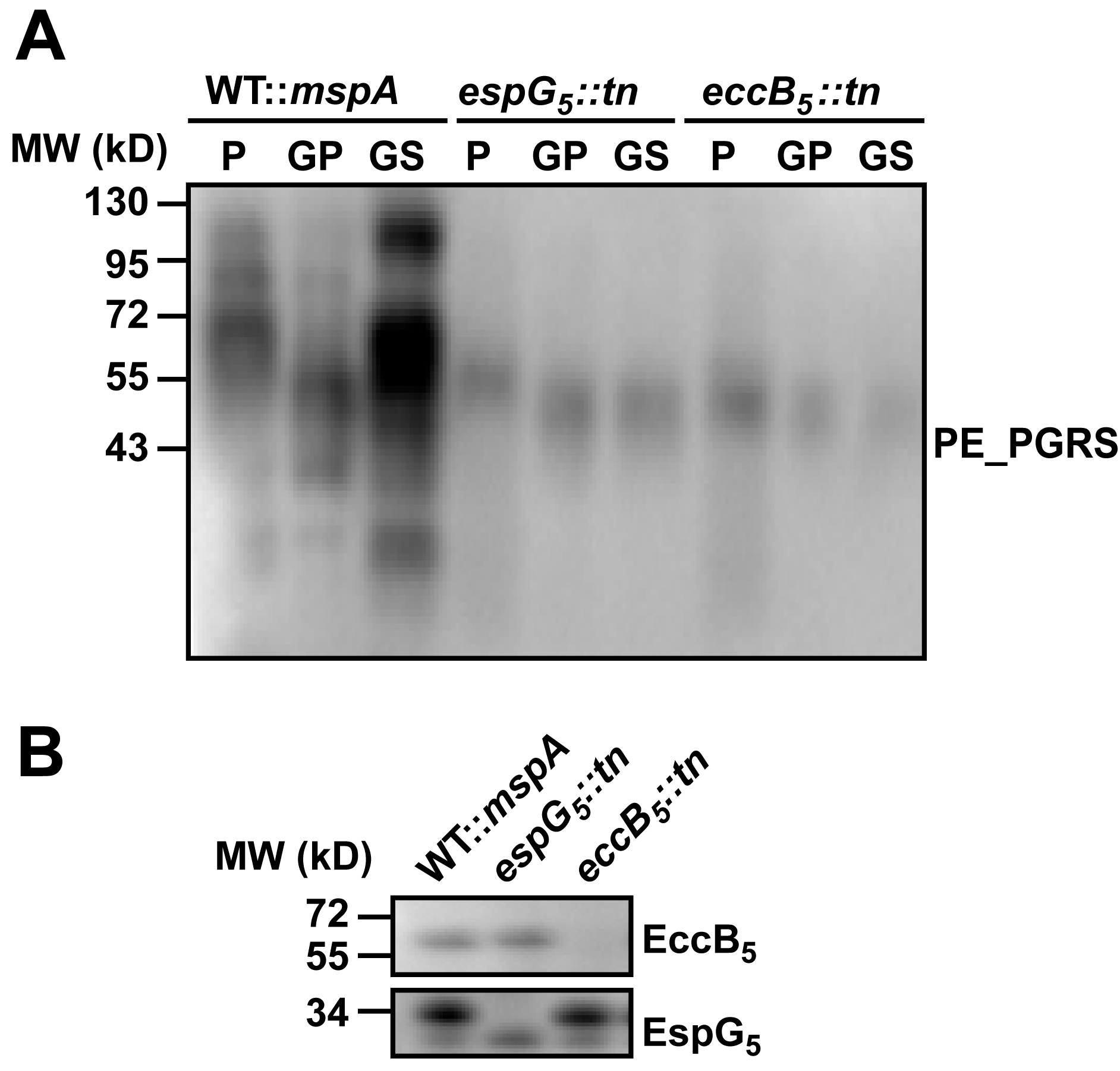

Supplement: S3 Fig — M. marinum::mspA and transposon mutants LA1 (espG 5::tn) and LA2 (eccB 5::tn) were analyzed for their expression and secretion of PE-PGRS proteins (A) and expression of EccB5 and EspG5 (B) by immunoblotting. Bacterial pellets were incubated with Genapol X-080, after which non-extracted material (GP) was separated by the solubilized material (GS) by centrifugation. Non-treated bacterial pellets (P) were additionally analyzed for the expression of EccB5 and EspG5 (B). (TIF) [file pgen.1005190.s003.tif]

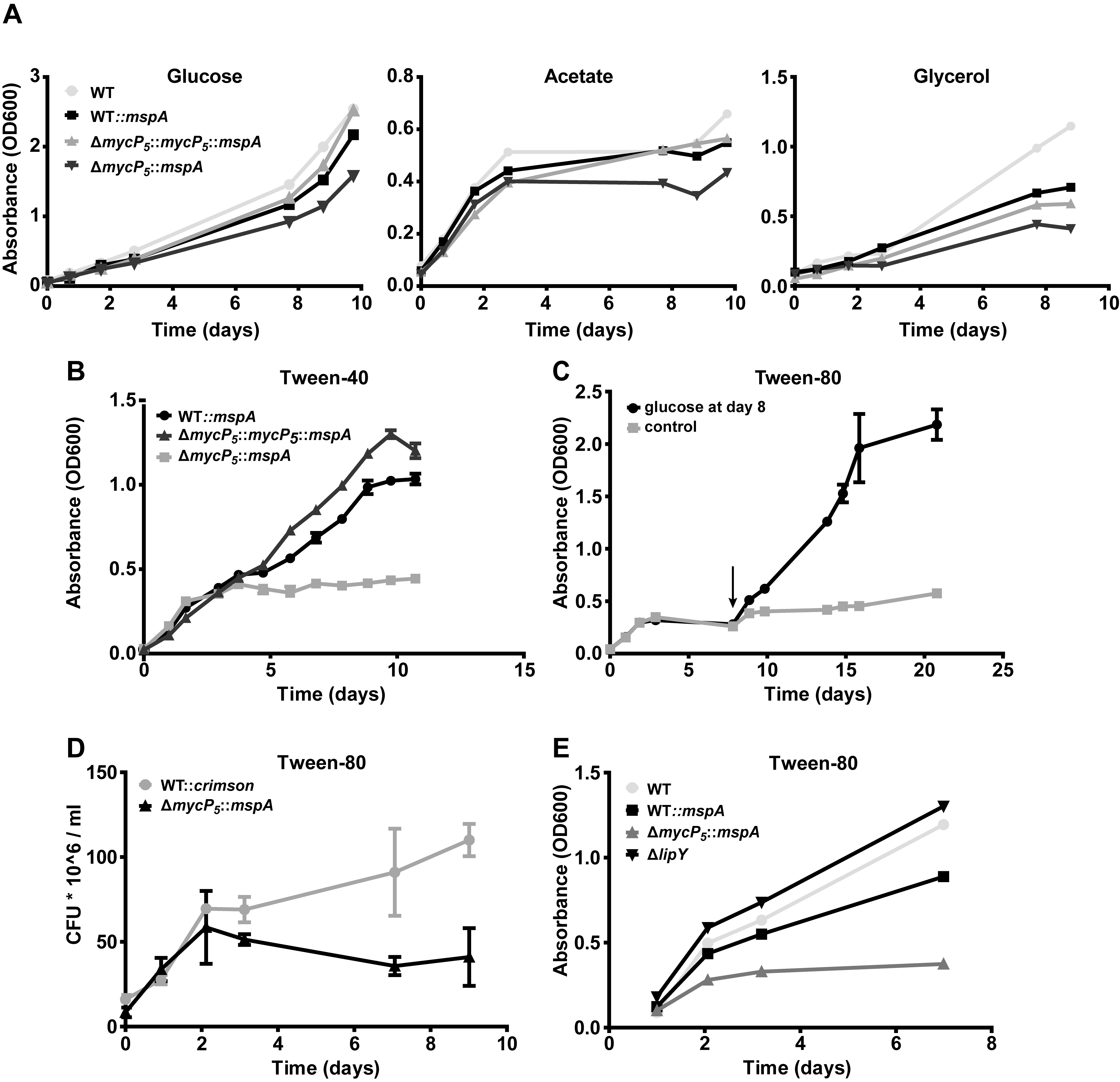

Supplement: S4 Fig — A) Growth of indicated M. marinum strains on 0.2% glucose, acetate or glycerol as a sole carbon source was assessed by measuring optical density at different time points. B) Growth of indicated strains at Tween-40. C) M. marinum ΔmycP5::mspA cannot grow on Tween-80 as a sole carbon source, but is able to grow normally when 0.2% glucose was added at day 8 (indicated by the arrow). D) M. marinum::pSMT3-crimson is not able to rescue growth of the mycP 5 mutant. A mixed culture of the two indicated strains on Tween-80 as a sole carbon source was performed. On the indicated time points bacteria were plated out on 7H10 plates with hygromycin and colony forming units (CFU) were quantified by counting fluorescent (WT) and non-fluorescent (ΔmycP 5) colonies. E) M. marinum ΔlipY can grow normally on Tween-80 as a sole carbon source. In B-D, error bars depict the standard deviation over three independent cultures. (TIF) [file pgen.1005190.s004.tif]

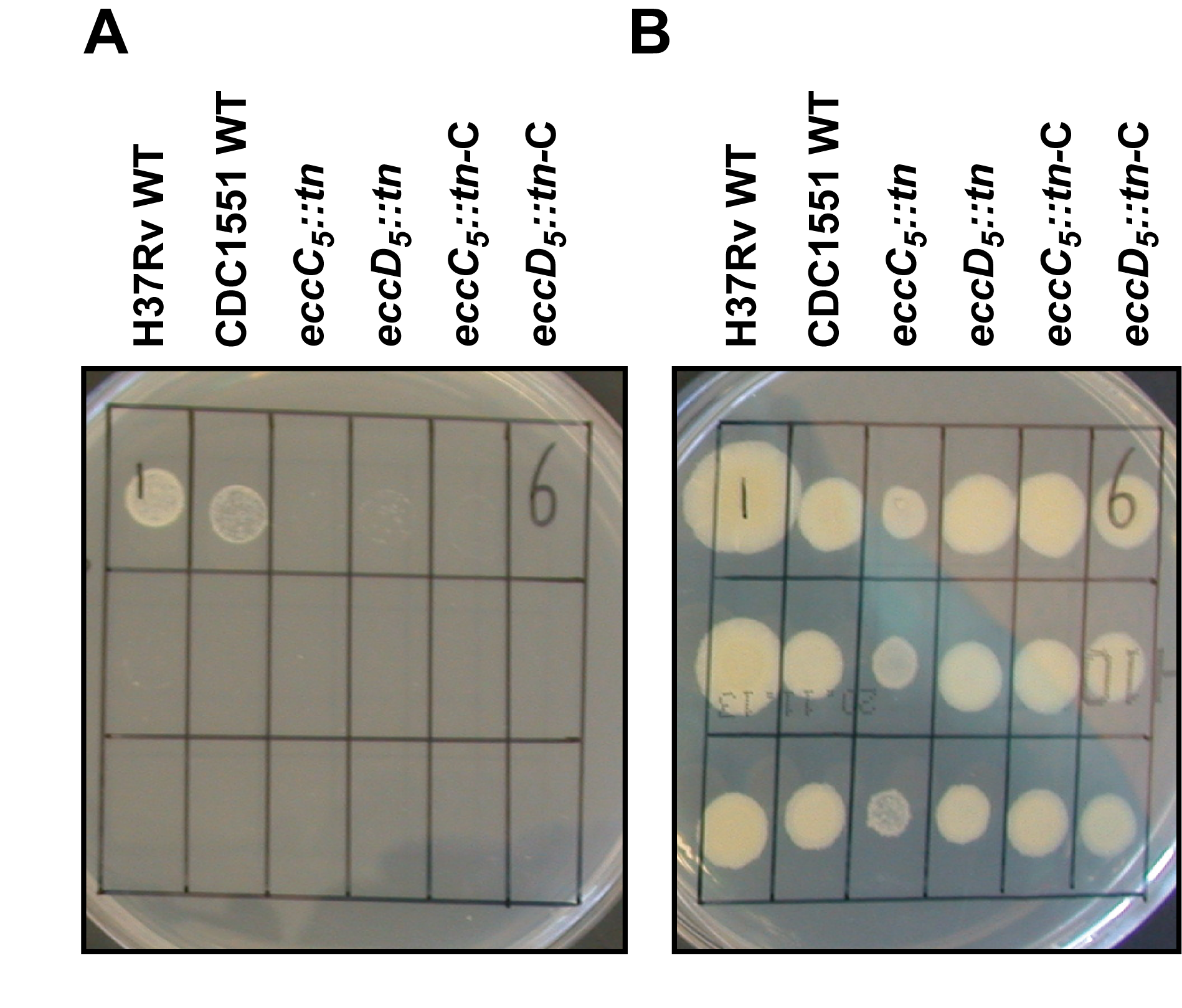

Supplement: S5 Fig — M. tuberculosis H37Rv and CDC1551 wild-type strains, CDC1551 derived transposon mutants in eccC 5 (eccC 5::tn) or eccD 5 (eccD 5::tn); and their respective complemented strains (eccC 5::tn-C and eccD 5::tn-C), were grown on 7H10 plates containing a combination of 30μg/ml ampicillin and 30μg/ml clavulanic acid (A), or without antibiotics (B). 5 μl of a 1.0 OD600 culture was spotted in serial dilutions (top row, undiluted; second row, 10x diluted; third row 100x diluted). In the absence of antibiotics, the H37Rv strain grew faster than the CDC1551 strains, while the eccC 5::tn strain showed a growth defect, which was alleviated upon complementation. Both ESX-5 transposon mutants and their respective complementants did not grow in the presence of 30μg/ml ampicillin and 30μg/ml clavulanic acid, indicating increased membrane permeability in these strains compared to the wild-type strain. (TIF) [file pgen.1005190.s005.tif]

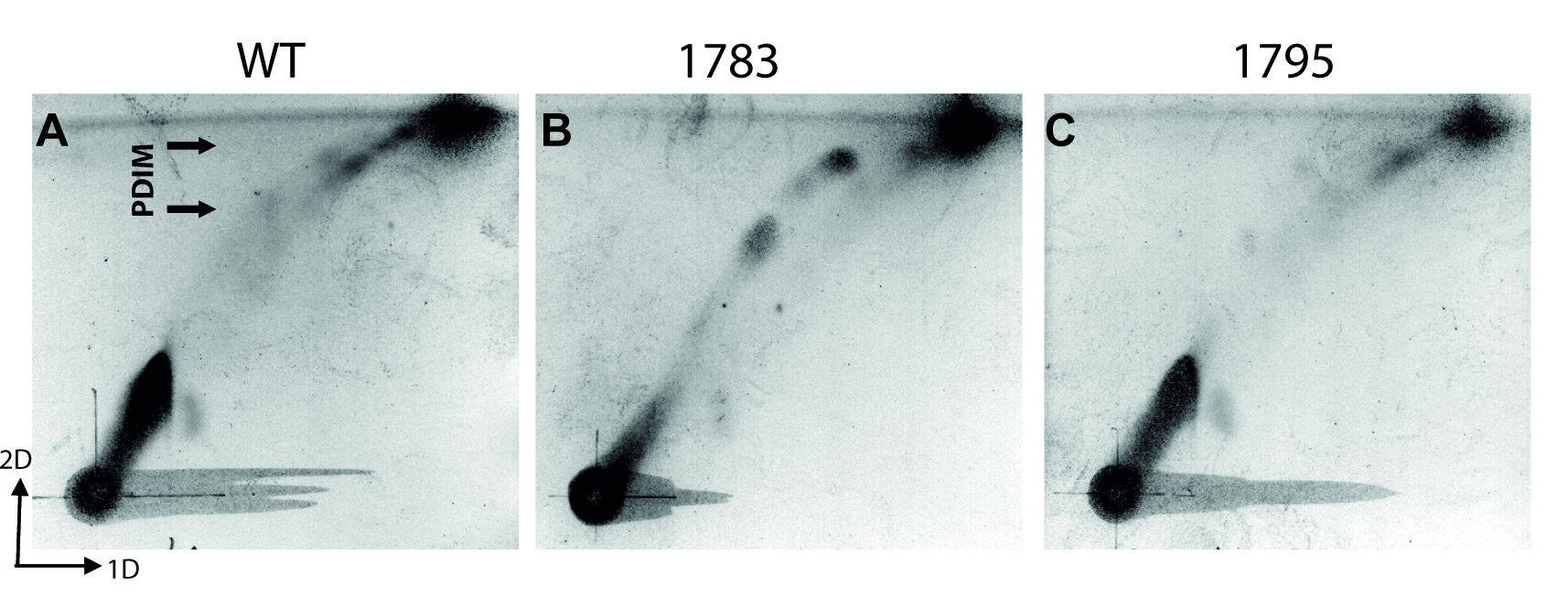

Supplement: S6 Fig — Apolar lipids were extracted from M. tuberculosis CDC1551 (A), and transposon mutants eccC 5::tn (rv1783, B) and eccD 5::tn (rv1795, C) as described before and were analyzed for the presence of PDIMs by 2D-TLC. 1D = PE:ethyl ether 98:2, 2D = PE:acetone 98:2. PDIMs are indicated by black arrows. PDIMs are visible in all strains, although the eccC 5 mutant appears to make higher amounts of PDIMs. (TIF) [file pgen.1005190.s006.tif]

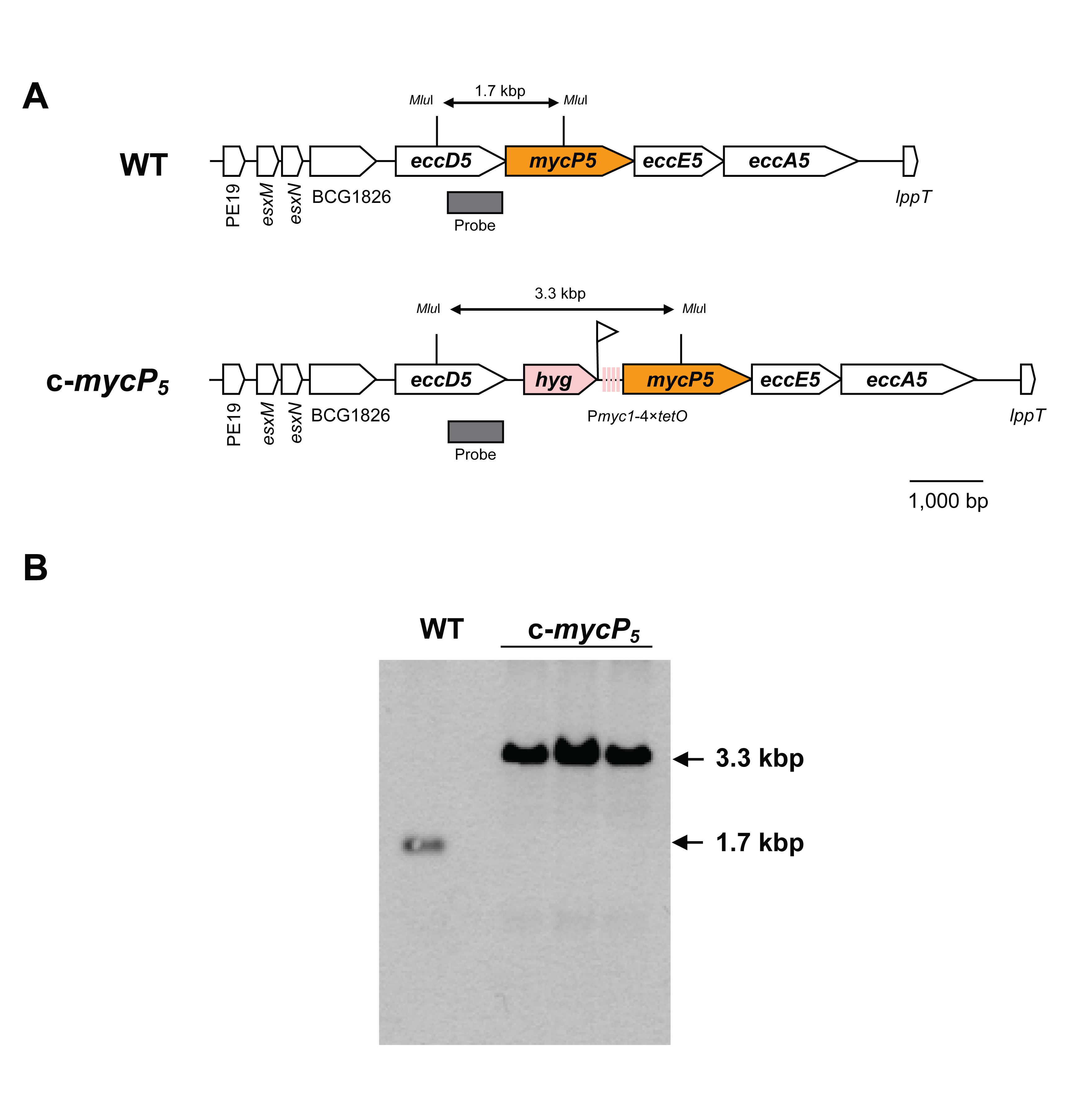

Supplement: S7 Fig — Construction of the M. bovis BCG-Pasteur c-mycP 5-Tet-on mutant. A) Organization of the mycP 5 locus in M. bovis BCG-Pasteur wild-type (WT) and the mycP 5 knock-in mutant. The sizes of relevant fragments as well as the location of the probe used for Southern blot analysis are indicated. Hyg, hygromycin resistance gene; Pmyc1-4×tetO, modified Pmyc1 promoter harboring 4 tetO sites. The same method was used to construct c-mycP 5-Tet-off, for which 4 synthetic tet-repressor sites (tetR) were introduced instead of the tetO sites. B) Southern blot analysis of MluI-digested genomic DNA using a probe hybridizing to the position indicated in (A), showing knock-in of the promoter cassette in c-mycP 5-Tet-on. (TIF) [file pgen.1005190.s007.tif]
