# Supplementary material for: Essential Role of the ESX-5 Secretion System in Outer Membrane Permeability of Pathogenic Mycobacteria
Source: PLoS Genet. 2015 May 4;11(5):e1005190. doi: 10.1371/journal.pgen.1005190 (PMC4418733; doi:10.1371/journal.pgen.1005190)
Supplement: S1 Table — Cell envelope proteins of M. marinum::mspA, Δesx-5::mspA and esx-5 tub strains were analyzed by LC-MS/MS. Proteins were classified as ESX-5-dependent when the normalized spectral counts between M. marinum::mspA and Δesx-5::mspA were reduced at least 10-fold. Grey highlighted rows indicate the conserved ESX-5 components. (DOCX) [file pgen.1005190.s008.docx]

**Table S1. ESX-5-dependent cell envelope proteins of *M. marinum*.** Cell envelope proteins of *M. marinum::mspA*, *Δesx-5::mspA* and *esx-5_tub_* strains were analyzed by LC-MS/MS. Proteins were classified as ESX-5-dependent when the normalized spectral counts between *M. marinum::mspA* and *Δesx-5::mspA* were reduced at least 10-fold. Grey highlighted rows indicate the conserved ESX-5 components.

|  | | MS/MS Normalized spectral counts^#^ | | | Fold Change |
| --- | --- | --- | --- | --- | --- |
| Identified protein | Functional description | *M.marinum ::mspA* | Δ*esx-5*  *::mspA* | Δ*esx-5*  *::esx-5_tub_* | wild-type/  Δ*esx-5::mspA* |
| **MMAR_2676** | EspG_5_ | 17 | 0.0 | 9.8* | ∞ |
| **MMAR_2680** | EccA_5_ | 12 | 0.0 | 2.3* | ∞ |
| **MMAR_1442** | PE_PGRS | 8.8 | 0.0 | 0 | ∞ |
| **MMAR_3797** | Lipid metabolism | 8.5 | 0.0 | 0 | ∞ |
| **MMAR_1129** | PPE | 8.5 | 0.0 | 0 | ∞ |
| **CtpA_1** | Cation transporter | 7.5 | 0.0 | 0.3 | ∞ |
| **PapA3** | Lipid metabolism | 6.5 | 0.0 | 0 | ∞ |
| **FadD9** | Lipid metabolism | 6.2 | 0.0 | 1.3 | ∞ |
| **MMAR_3408** | Oxidoreductase | 6.2 | 0.0 | 2.6 | ∞ |
| **CobN** | Cobalamin biosynthesis | 5.2 | 0.0 | 1.6 | ∞ |
| **MMAR_2679** | EccE_5_ | 45 | 0.8 | 19* | 59 |
| **FadD26** | Lipid metabolism | 12 | 0.3 | 1.3 | 34 |
| **MMAR_2677** | EccD_5_ | 60* | 2.2 | 36 | 27 |
| **MMAR_1790** | Cons. Hypothetical | 9.8 | 0.4 | 1.6 | 26 |
| **MetE** | Methionine biosynthesis | 7.9 | 0.4 | 2.0 | 26 |
| **MMAR_2664** | EccB_5_ | 40* | 2.1 | 35* | 21 |
| **MMAR_2665** | EccC_5_ | 162* | 12 | 132* | 19 |
| **LeuC** | Leucine biosynthesis | 8.5 | 0.7 | 5.9 | 13 |
| **SigB** | Sigma factor B | 8.1 | 0.8 | 2.3 | 12 |
| **LeuS** | Leucine tRNA ligase | 7.2 | 0.7 | 5.9 | 11 |
| **OtsB1** | Trehalose Biosynthesis | 7.5 | 0.7 | 2.6 | 11 |

* Spectral counts for *M. marinum* and *M. tuberculosis* orthologues were combined into one value.

# Spectral counts were normalized in a two-way analysis between *M.marinum*::*mspA* and Δe*sx-5::mspA*, or in a two-way analysis between *M. marinum* and Δe*sx-5::esx-5_tub_*. Data for wild-type *M. marinum* are not depicted.
