# Supplementary material for: Essential Role of the ESX-5 Secretion System in Outer Membrane Permeability of Pathogenic Mycobacteria
Source: PLoS Genet. 2015 May 4;11(5):e1005190. doi: 10.1371/journal.pgen.1005190 (PMC4418733; doi:10.1371/journal.pgen.1005190)
Supplement: S2 Table — (DOCX) [file pgen.1005190.s009.docx]

**Table S2.** List of primers used for cloning in this study.

| **Name** | **Sequence (5’ 🡪 3’)** |
| --- | --- |
| EccC ko Lf | TTTTTTTTCCATAAATTGGGCCGTCGGACTTCAATCCAG |
| EccC ko Lr | TTTTTTTTCCATTTCTTGGCGGTGTTGGCAGAACGATGT |
| EccC ko Rf | TTTTTTTTCCATAGATTGGTCATTCGCGGCAAGATGAAG |
| EccC ko Rr | TTTTTTTTCCATCTTTTGGGAATGGCCACTACTACCTGA |
| MycP5 LF | TTTTTTTTCAGAAACTGATCCTTGCCTCGGGCGTCTT |
| MycP5 LR | TTTTTTTTCAGTTCCTGCGCCACGGCCAAATTGCCAT |
| MycP5 RF | TTTTTTTTCAGAGACTGAGGAATCGGCTCATTCACAC |
| MycP5 RR | TTTTTTTTCAGCTTCTGCAATATCGCGCTGTCAACCT |
| EccB5MunI.fw | GATGCAATTGCATATGGCTGAACAAGGCCGCGGA |
| EccC5HindIII.rev | GATGAAGCTTTTACTTTCGCACCTCGGTCA |
| EccB stop 1 | CGCGTTATGAATT |
| EccB stop 2 | CGCGAATTCATAA |
| EccC stop 1 | CATGTTATGAATT |
| EccC stop 2 | CATGAATTCATAA |
| BC Van91I F | GACGACGCCAAGGAGTACAA |
| BC Van91I R | AAGATCTCCGGACCACTCCA |
| BC NcoI F | CGAGAAGGCGCCGGTAATCA |
| BC XmnI R | CTGCCGCGAACCGAGTTGTA |
| BC EcoRV R | GGCATGGTCGCGGTCAACTA |
| BC K506A F | GTACCACGGGTTCCGGTGCCTCGACGCTGGTGCGGACCGT |
| BC K506A R | ACGGTCCGCACCAGCGTCGAGGCACCGGAACCCGTGGTAC |
| BC K879A F | GGGTGGATCGGGCGCCACGACCGCGCTGCAGACACTGATT |
| BC K879A R | AATCAGTGTCTGCAGCGCGGTCGTGGCGCCCGATCCACCC |
| BC R1181A F | GGCCGGCGCGAATGTGGGGCCACCACAACGTTGGCCACGA |
| BC R1181A R | TCGTGGCCAACGTTGTGGTGGCCCCACATTCGCGCCGGCC |
| BC R1181K F | GGCCGGCGCGAATGTGGGAAGACCACAACGTTGGCCACGA |
| BC R1181K R | TCGTGGCCAACGTTGTGGTCTTCCCACATTCGCGCCGGCC |
| BC R1365 stop R | GATAAGCTTCAAGGCAGCGGGCCACCCTTC |
| MAS HindIII F | CTTAAGCTTCCTACTCCGCAGCAGCGTCA |
| MAS R | TTGTGCAGATCGGCTCTCGT |
| md_MMAR2678_F2 | CCGGAATTCCATATGCAGCGATTCGGTACCGTT |
| md_MMAR2678_R | GCCGAAGCTTTCATCGCCGCTTCCGTGA |
| MycP5S461A.fw | GCTGGCACGGCCTTTGCGACGG |
| MycP5S461A.rev | CCGTCGCAAAGGCCGTGCCAGC |
| MycP5dTM.rev | GCCGAAGCTTTCAATTGCGCTCTGCGGGCGG |
| MspA-NheI-F | GCACGCTAGCATGAAGGCAATCAGTCGGGT |
| MspA-BamHI-R | CCCGGATCCTCAGTTCATGTTCCAGGGTT |
| pMyco1 | CCGGGGACTTATCAGCCAAC |
| T7_1 | CCCGAAAAGTGCCACCTAAATTGTAAGCG |
| T7_2 | CGCTTCCTCGTGCTTTACGGTATCGCC |
| pSalg | GCTTATTCCTCAAGGCACGA |
| MycP5KIUpFw | TTTTTCACAAAGTGCATCATCGGTGCGCTGACAATGC |
| MycP5KIUpRv | TTTTTCACTTCGTGCTACCGGTACCGAATCGCTGC |
| MycP5KIDownFw | TTTTTCCATAGATTGGATGCAGCGATTCGGTACCGGTAG |
| MycP5KIDownRv | TTTTTCCATCTTTTGGCCGGAGTAGCTGGGTACTGTCAC |
| TetRFw | TTTTTTGAATTCATGATGTCTAGATTAGATAAAAG |
| TetRRv | TTTTTTAAGCTTAAGACCCACTTTCACATTTAAG |
| RevTetRFw | TTTTTGAATTCATGAGCACGATCCGCGGTACCATC |
| RevTetRRv | TTTTTAAGCTTAGGAGCCGCTCTCGCACTTCAG |
| Exs-5 ko Lf | TTTTTTTTCCATAAATTGGATCGCCTCGACACGAAGGTA |
| Esx-5 ko Lr | TTTTTTTTCCATTTCTTGGCGGCCTTGTTCAGCCACGTTA |
| Esx-5 ko Rf | TTTTTTTTCCATAGATTGGGACCTGGCCGAAGGTCTCAGT |
| Esx-5 ko Rr | TTTTTTTTCCATCTTTTGGCGGCGATCACCGTCTCGTAT |
