# Supplementary material for: Essential Role of the ESX-5 Secretion System in Outer Membrane Permeability of Pathogenic Mycobacteria
Source: PLoS Genet. 2015 May 4;11(5):e1005190. doi: 10.1371/journal.pgen.1005190 (PMC4418733; doi:10.1371/journal.pgen.1005190)
Supplement: S3 Table — (DOCX) [file pgen.1005190.s010.docx]

**Table S3.** Primers used for TraDIS experiments.

| **Name** | **Adapter** |
| --- | --- |
| Ind_Ad_T | ACACTCTTTCCCTACACGACGCTCTTCCGATC*T |
| Ind_AD_B | pGATCGGAAGAGCGGTTCAGCAGGAATGCCGAGACCGATCTC |
|  |  |
|  | **Primers for qPCR** |
| TopA_qPCR | CTTTCCCTACACGACGCTCTTC |
| BotA_qPCR | ATTCCTGCTGAACCGCTCTTC |
|  |  |
|  | **qPCR for PCR - amplified libraries** |
| Syb_FP5 | ATGATACGGCGACCACCGAG |
| Syb_RP7 | CAAGCAGAAGACGGCATACGAG |
|  |  |
|  | **Mycomar P5 primers** |
| Mycomar_P5_PCR3pr1 | AATGATACGGCGACCACCGAGATCTACAC TCTGGGGTACGCGTAATACGACTC |
| Mycomar_3pr_seq | CACTATAGGATCTAGAGACCGGGGACTT |
| Mycomar_p5_PCR5pr1 | AATGATACGGCGACCACCGAGATCTACACAGGGTTGAGTGTTG  TTCCAGTTTG |
| Mycomar_5pr_seq | CTATAGGGATCTAGAGACCGGGGACTTA |

|  | **Transposon index read primer** |
| --- | --- |
| RInvSeq | AGATCGGAAGAGCGTCGTGTAGGGAAAGAGTGT |

|  | **Multiplexing primers RInv4** |
| --- | --- |
| RInV 4.1 | CAAGCAGAAGACGGCATACGAGATCGGTACAAGCTA  ACACTCTTTCCCTACACGACGCTCTTCCGATCT |
| RInV 4.2 | CAAGCAGAAGACGGCATACGAGATCGGTAAACATCG  ACACTCTTTCCCTACACGACGCTCTTCCGATCT |
| RInV 4.3 | CAAGCAGAAGACGGCATACGAGATCGGTACATTGGC  ACACTCTTTCCCTACACGACGCTCTTCCGATCT |
| RInV 4.4 | CAAGCAGAAGACGGCATACGAGATCGGTACCACTGT  ACACTCTTTCCCTACACGACGCTCTTCCGATCT |
| RInV 4.5 | CAAGCAGAAGACGGCATACGAGATCGGTAACGTGAT  ACACTCTTTCCCTACACGACGCTCTTCCGATCT |
| RInV 4.6 | CAAGCAGAAGACGGCATACGAGATCGGTCGCTGATC  ACACTCTTTCCCTACACGACGCTCTTCCGATCT |
| RInV 4.7 | CAAGCAGAAGACGGCATACGAGATCGGTCAGATCTG  ACACTCTTTCCCTACACGACGCTCTTCCGATCT |
| RInV 4.8 | CAAGCAGAAGACGGCATACGAGATCGGTATGCCTAA  ACACTCTTTCCCTACACGACGCTCTTCCGATCT |
| RInV 4.9 | CAAGCAGAAGACGGCATACGAGATCGGTCTGTAGCC  ACACTCTTTCCCTACACGACGCTCTTCCGATCT |
| RInV 4.10 | CAAGCAGAAGACGGCATACGAGATCGGTAGTACAAG  ACACTCTTTCCCTACACGACGCTCTTCCGATCT |
| RInV 4.11 | CAAGCAGAAGACGGCATACGAGATCGGTCATCAAGT  ACACTCTTTCCCTACACGACGCTCTTCCGATCT |
| RInV 4.12 | CAAGCAGAAGACGGCATACGAGATCGGTAGTGGTCA  ACACTCTTTCCCTACACGACGCTCTTCCGATCT |
