# Supplementary material for: Essential Role of the ESX-5 Secretion System in Outer Membrane Permeability of Pathogenic Mycobacteria
Source: PLoS Genet. 2015 May 4;11(5):e1005190. doi: 10.1371/journal.pgen.1005190 (PMC4418733; doi:10.1371/journal.pgen.1005190)
Supplement: S4 Table — (DOCX) [file pgen.1005190.s011.docx]

**Table S4.** List of plasmids used in this study.

| **Name** | **Features** | **Source** |
| --- | --- | --- |
| pMV361-*hyg* | *hsp60* promotor, L5 *attP* & *aph, oriE, hyg*^R^ | (49) |
| pMV-*eccBC_5_-kan* | *hsp60* promotor, L5 *attP* & *aph, oriE, kan*^R^ | This study |
| pMV-*mycP_5_-kan* | *hsp60* promotor, L5 *attP* & *aph, oriE, kan*^R^ | This study |
| pMV-*eccBC_5_-hyg* | *hsp60* promotor, L5 *attP* & *aph, oriE, hyg*^R^ | This study |
| pMV-*mycP_5_-hyg* | *hsp60* promotor, L5 *attP* & *aph, oriE, hyg*^R^ | This study |
| pMV-*eccB_5_ P145 stop* | *hsp60* promotor, L5 *attP* & *aph, oriE, hyg*^R^ | This study |
| pMV-*eccC_5_ V57 stop* | *hsp60* promotor, L5 *attP* & *aph, oriE, hyg*^R^ | This study |
| pMV-*eccC_5_ R1365stop* | *hsp60* promotor, L5 *attP* & *aph, oriE, hyg*^R^ | This study |
| pMV-*eccC_5_* K506A (NBD1) | *hsp60* promotor, L5 *attP* & *aph, oriE, hyg*^R^ | This study |
| pMV-*eccC_5_* K879A (NBD2) | *hsp60* promotor, L5 *attP* & *aph, oriE, hyg*^R^ | This study |
| pMV-*eccC_5_* R1181A (NBD3) | *hsp60* promotor, L5 *attP* & *aph, oriE, hyg*^R^ | This study |
| pMV-*eccC_5_* R1181K (NBD3) | *hsp60* promotor, L5 *attP* & *aph, oriE, hyg*^R^ | This study |
| pMV-*mas* | *hsp60* promotor, L5 *attP* & *aph, oriE, hyg*^R^ | This study |
| pMV-*eccBC_5_stop-mas* | *hsp60* promotor, L5 *attP* & *aph, oriE, hyg*^R^ | This study |
| pMV-*eccBC_5_-mas* | *hsp60* promotor, L5 *attP* & *aph, oriE, hyg*^R^ | This study |
| pMV-*esx5*_tub_ | *hsp60* promotor, L5 *attP* & *aph, oriE, hyg*^R^ | This study |
| pSMT3-*mspA* | *hsp60* promotor, pAL5000 origin, *oriE, hyg*^R^ | This study |
| pUCintCat-*mycP_5_* | *ag85* promotor, L5 *attP* & *aph, oriE, cat*^R^ | This study |
| pSM128 | *hsp60* promotor, L5 *attP* & *aph, oriE, strep*^R^ | (51) |
| pMV261::*tetR*-RBS-E | *hsp60* promotor, *OriM*, *tetR* | This study |
| pMV261::*rev-tetR*-RBS-F | *hsp60* promotor, *OriM*, *rev-tetR* | This Study |
